# pH Dependent Reversible Formation of a Binuclear Ni_2_ Metal-Center Within a Peptide Scaffold

**DOI:** 10.3390/inorganics7070090

**Published:** 2019-07-16

**Authors:** Brenna C. Keegan, Daniel Ocampo, Jason Shearer

**Affiliations:** Department of Chemistry, Trinity University, 1 Trinity Place, San Antonio, TX 78212, U.S.A.

**Keywords:** biological nickel sites, nickel-thiolates, dinuclear nickel metallopeptides, thiolate oxidative damage

## Abstract

A disulfide-bridged peptide containing two Ni^2+^ binding sites based on the nickel superoxide dismutase protein, {Ni_2_(SOD^mds^)}, has been prepared. At physiological pH (7.4) it was found that the metal sites are mononuclear with a square planar NOS_2_ coordination environment with the two sulfur-based ligands derived from cysteinate residues, the nitrogen ligand derived from the amide backbone and a water ligand. Furthermore, S K-edge X-ray absorption spectroscopy indicated that the two cysteinate sulfur atoms ligated to nickel are each protonated. Elevation of the pH to 9.6 results in the deprotonation of the cysteinate sulfur atoms, and yields a binuclear, cysteinate bridged Ni_2_^2+^ center with each nickel contained in a distorted square planar geometry. At both pH = 7.4 and 9.6 the nickel sites are moderately air sensitive, yielding intractable oxidation products. However, at pH = 9.6 {Ni_2_(SOD^mds^)} reacts with O_2_ at an ~3.5-fold faster rate than at pH = 7.4. Electronic structure calculations indicate the reduced reactivity at pH = 7.4 is a result of a reduction in S(3p) character and deactivation of the nucleophilic frontier molecular orbitals upon cysteinate sulfur protonation.

## 1. Introduction

**Chart 1.**
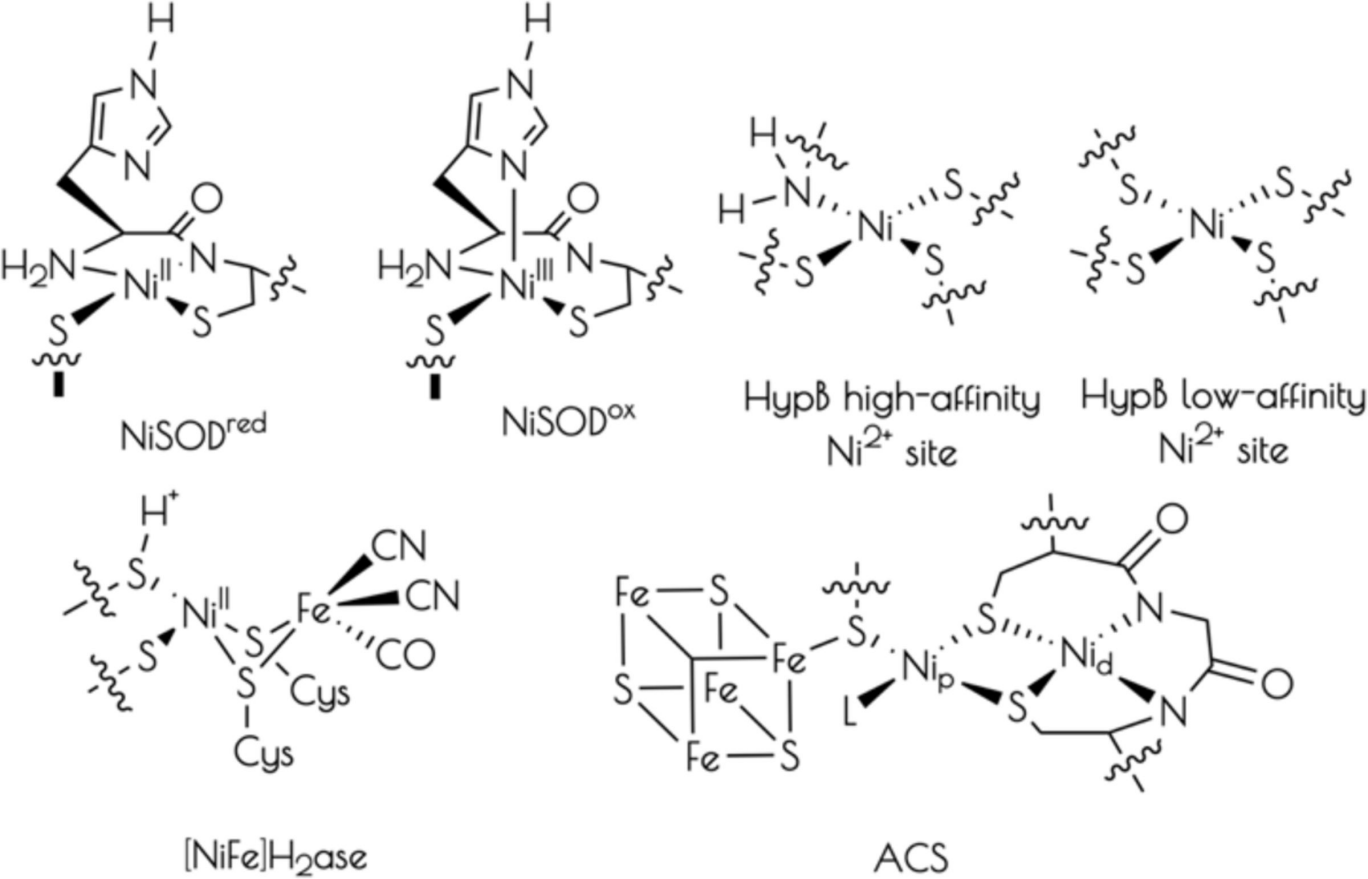
Representative structures of the active sites of cysteinate-ligated nickel containing metalloproteins.

Nickel is an essential biological co-factor found at the active-sites of a number of microbial metalloenzymes and proteins (Chart 1).^1–5^ Broadly divided into redox active and non-active nickel metalloproteins, it has been recognized that the majority of known redox active nickel containing metalloenzymes contain cysteinate sulfur ligation to nickel. Cysteinate ligation appears necessary to poise nickel-based one-electron redox couples so as to be accessible under physiological conditions. It has also been demonstrated that several redox inactive nickel transport and regulatory proteins also possess cysteinate ligands to Ni^2+^.^6–11^

An interesting feature of the nickel-thiolate moiety is its ability to support ligand protonation without subsequent protonolysis.^12–14^ This is most often observed in (near) square planar Ni^2+^ centers where the nucleophilic HOMO possesses significant S(3pπ) character, which effectively act as S-based lone-pairs. To date, two nickel containing metalloenzymes, nickel iron hydrogenase [NiFe]H_2_ase and nickel containing superoxide dismutase (NiSOD), have been demonstrated to possess at least one cysteinate ligand that becomes protonated, forming a Ni-S(H^+^)-Cys moiety under physiological conditions.^15–16^ Concerning the role of the Ni-S(H^+^)-Cys moiety in biochemical reactions, it has been proposed that these moieties can behave as proton donors/acceptors and sources of formal hydrogen atoms.^17–22^ However, their exact role(s) in biological reactions is currently unknown.

NiSOD is a nickel containing homohexameric metalloenzyme that disproportionates O_2_^−^ into H_2_O_2_ and O_2_ by cycling between reduced Ni^2+^ and oxidized Ni^3+^ oxidation states.^23–25^ Each monomer contains a mononuclear nickel site that is coordinated by two *cis*-cysteinate sulfur atoms from Cys2 and Cys6, an amidate nitrogen atom from Cys2, and the *N*-terminal amine nitrogen atom from His1 (Chart 1). Upon oxidation to Ni^III^, the square planar nickel site ligates the N^ε^ imidazole from His1 forming a square pyramidal coordination geometry. Taking advantage of the fact that all of the ligating residues to nickel are found within the first six residues from the protein *N*-terminus, we, and others, have prepared functional NiSOD metallopeptide based mimics utilizing the first 6 – 12 residues from the NiSOD *N*-terminal primary protein sequence. These metallopeptide based mimics reproduce the key structural and spectroscopic properties of the metalloenzyme.^17, 26–33^

As stated above, NiSOD itself has been shown to possess at least one Ni-S(H^+^)-Cys moiety in its reduced form.^15^ Although not without controversy,^33^ we have provided strong evidence based on sulfur K-edge X-ray absorption studies that like NiSOD itself, NiSOD metallopeptide based mimics possess a Ni-S(H^+^)-Cys moiety at physiological pH as well.^18, 22^ Studies have also suggested that the p*K*_*a*_ of the Ni-S(H^+^)-Cys proton within these active-sites is ~8.5, and can become reversibly deprotonated at high pH (> 9.0).22

In addition to mimicking the NiSOD active site, derivatives of the NiSOD inspired metallopeptides are capable of not only mimicking NiSOD, but also mimicking the active-site of cobalt containing NHase, and coordinating Cu^2+^.^34–36^ This inspired us to further derivatize the a NiSOD metallopeptide mimic, SOD^m1^ (SOD^m1^ = (SOD^mds^ = HCDLP-CGVYDA-PA), in order to generate different metal-site structures. The intent of such studies is not necessarily to generate NiSOD biomimetic metallopeptides, but instead to probe different metal coordination environments within a biologically derived scaffold. Herein, we present work on a nickel metallopeptide, {Ni_2_^II^(SOD^mds^)} (SOD^mds^ = (T^a^CDLP-CGVYDA-PA)_2_, where T^a^ is a 2-mercaptoacetate group). It will be demonstrated that at pH = 7.4 this metallopeptide possess two mononuclear Ni^2+^ sites that support the formation of the Ni-S(H^+^)-Cys moiety. Furthermore, we will show that the metallopeptide forms a dinuclear cysteinate bridged Ni_2_^2+^ center upon elevation of the pH to 9.6. Lastly, it will be demonstrated that Ni-S-Cys protonation protects the metallopeptide from oxidative damage by O_2_. The protection of the metallopeptide against oxidative damage will be rationalized in terms of an alteration of the electronic structure of the nickel-site upon protonation rendering the thiolate sulfurs relatively inert towards oxidation.

## 2. Results

### 2.1. Generation of {Ni_2_^II^(SOD^mds^)}.

**Figure 1.**
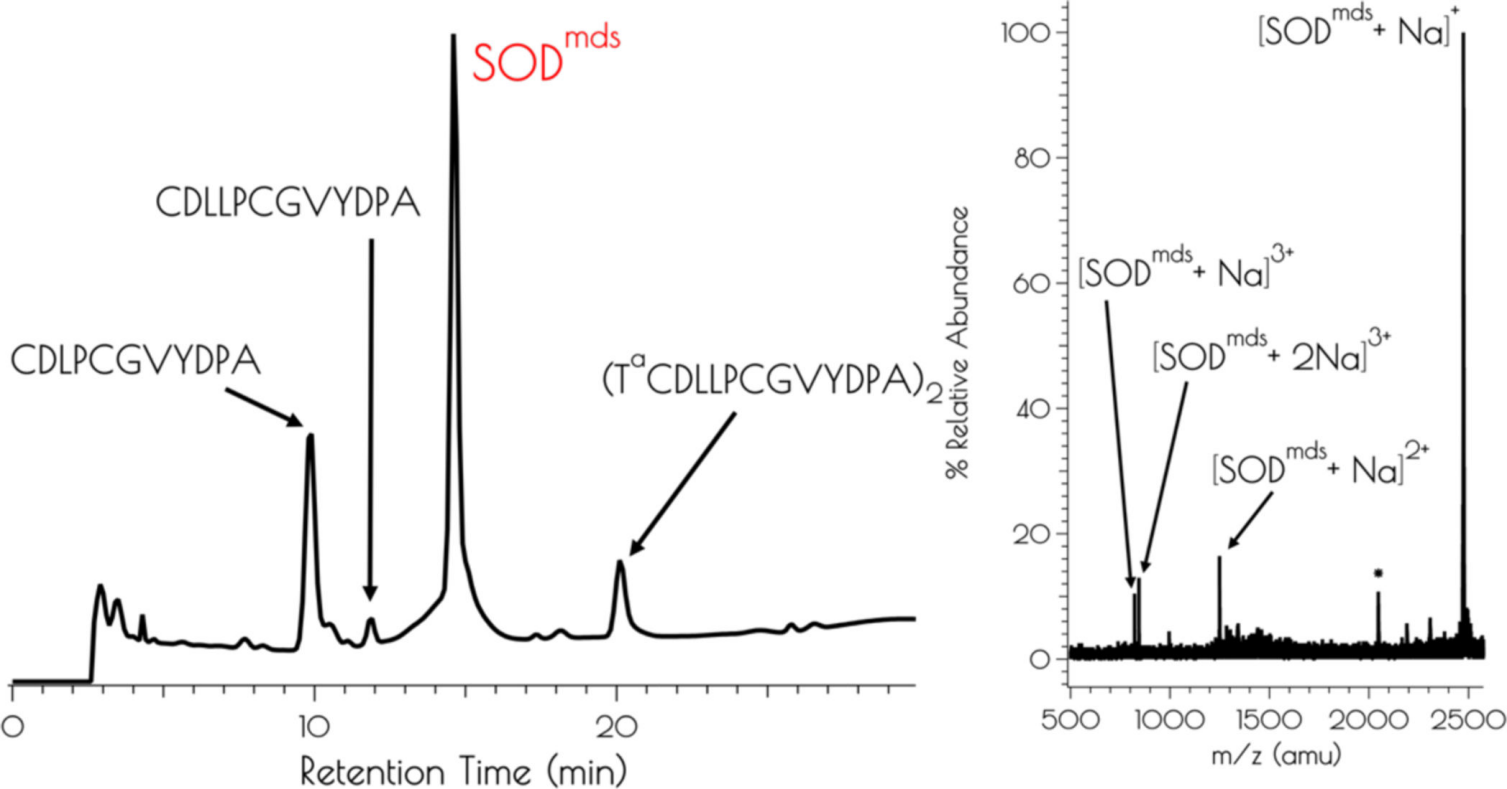
Left: Analytical HPLC chromatogram of the crude mixture resulting from the synthesis of SOD^mds^ with a detector cutoff of 0 intensity units. Identifiable products are highlighted. A mobile phase of a mixture of 0.1% TFA in water and 0.1% TFA in acetonitrile and a linear gradient of 9:1 water:acetonitrile − 4:6 water:acetonitrile over the course of 30 min. Right: MALDI-TOF of the purified peptide SOD^mds^ (* indicates [SOD^mds^]^+^ with the YDPA residues cleaved from the C-terminus of one of the monomers).

The apo-peptide SOD^mds^ (SOD^mds^ = (T^a^CDLP-CGVYDA-PA)_2_) was prepared by standard solid phase peptide synthesis using Fmoc/^*t*^Bu protection strategies. Cleavage of the peptide from the resin and subsequent global deprotection of the side-chain residues was effected by so-called reagent K. Reagent K is a peptide cleavage mixture that is used for peptides containing readily oxidizable residues such as Cys and Tyr, and contains a number of scavengers that dramatically reduce oxidative side reactions.^37^ Despite the use of reagent K, we found that the apo-peptide formed a disulfide bond upon cleavage from the resin; even workup under anaerobic conditions using rigorously purified diethyl ether yielded a crude peptide lacking monomeric disulfide free peptide as evidenced by mass spectrometry (MS) and an Ellman’s assay (Figure 1). Figure 1 depicts an HPLC chromatogram of the crude product. LCMS examination of all components of the reaction mixture demonstrated that no monomeric peptide containing the *N*-terminal 2-mercaptoacetate group was produced.

An Ellman’s assay^38^ demonstrated that SOD^mds^ possessed four free thiol groups per dimeric apopeptide. This is similar to what has been reported for all NiSOD metallopeptides generated to date. For these NiSOD inspired peptides lacking the *N*-terminal thiolate group, cleavage of the apo-peptide from the resin and subsequent *aerobic* work-up and purification yield purified monomeric peptides free of disulfide bonds between the two cysteinate sulfur atoms corresponding to Cys2 and Cys6 in the wild-type NiSOD sequence.^26–27, 30, 34^ Considering the only major modification between SOD^mds^ and similar NiSOD inspired peptides generated to date is the presence of the 2-mercaptoacetate group, we suggest that the disulfide moiety within SOD^mds^ results from the oxidative S-S bond formation between two *N*-terminal thiol groups from two different peptides. Furthermore, all identifiable monomeric peptides by LCMS resulting from incomplete/mis-coupling events lacked the *N*-terminal 2-mercaptoacetate group, lending further support of the *N*-terminal disulfide bond in SOD^mds^. This supposition will be shown to be further validated by computational modeling of the resulting nickel site (vide infra).

**Figure 2.**
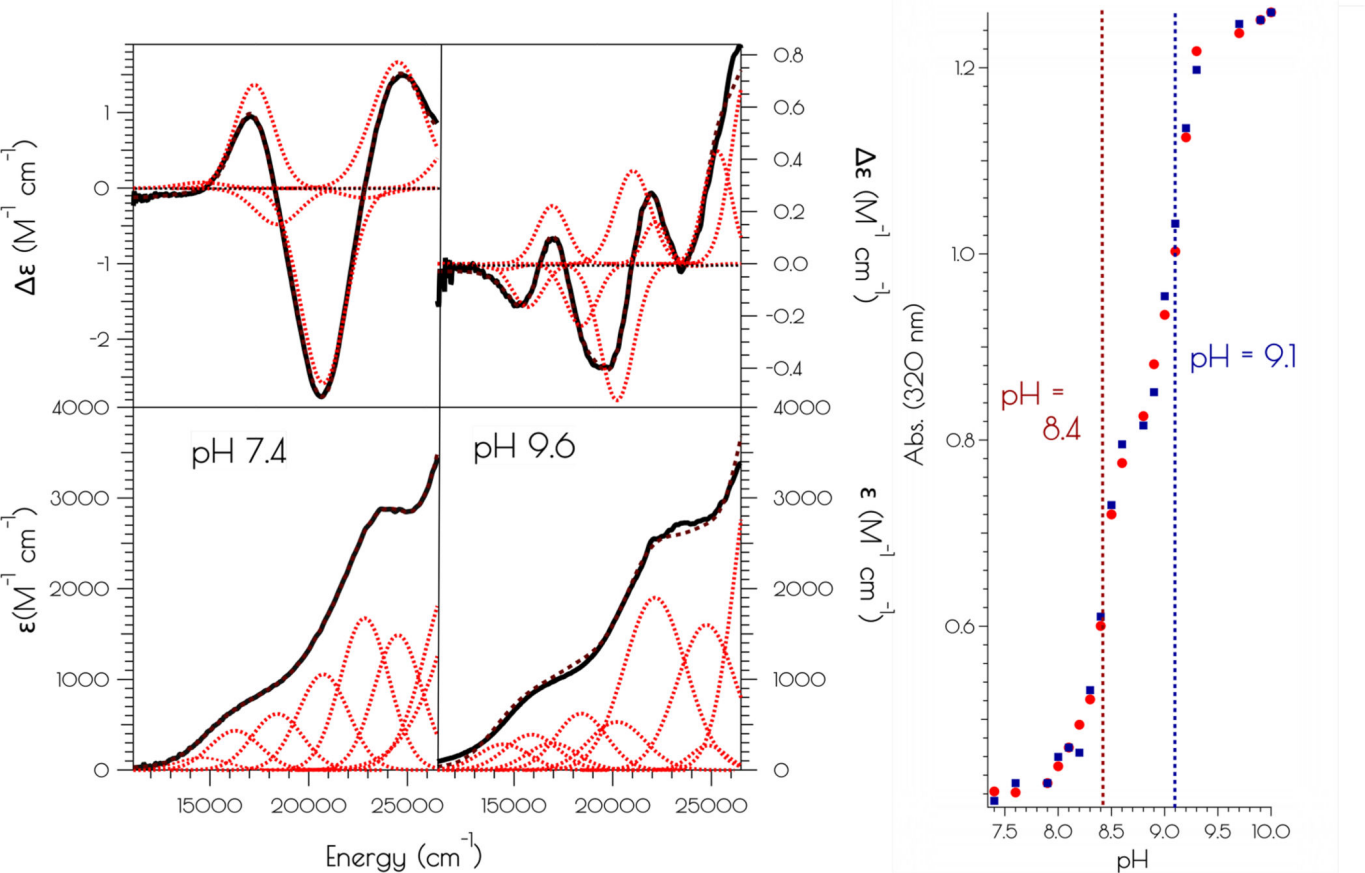
Left: electronic absorption spectra (bottom) and CD spectra (top) of {Ni_2_^II^(SOD^mds^)} at pH 7.4 and 9.6. The solid black spectra represent the experimental data, the red dashed curves represent the individual transitions deconvolved from the spectra and the dashed black spectra represent the convolution of the individual transitions. Right: pH profile showing the change in absorbance at 320 nm vs change in pH upon going from pH 7.4 to 9.6 (red circles) and pH 9.6 to 7.4 (blue squares).

The addition of two equivalents of NiCl_2_ to a pH 7.4 solution (50 mM *N*-ethylmorpholine, NEM) of SOD^mds^, forming {Ni_2_(SOD^mds^)}, yields a pinkish-tan solution. Raising the pH to 9.6 (50 mM NEM) causes a color change from the pinkish tan color to a more intensely colored reddish-brown solution. Although subtle changes in the far visible region of the electronic absorption spectra are noted at elevated solution pH, a much larger change is noted in the CD spectrum upon changing the solution pH from 7.4 to 9.6 (Figure 2). Monitoring the change in the electronic absorption spectrum vs the change in pH shows a significant increase in the absorbance at 320 nm with two inflection points in the pH profile at a pH of 8.4 and 9.1. The change in the spectra in response to a change in pH is fully reversible. This suggests that there are two protonatable sites within {Ni_2_(SOD^mds^)}. We note that the Y9 phenolic group will become deprotonated at the higher pH value, however, the change in absorbance at 320 nm upon deprotonation of phenol will be minimal and not dramatically influence the intensity of the electronic absorption spectrum at 320 nm. Thus, we do not attribute the second deprotonation/protonation event to Y9 deprotonation.

### 2.2. Nickel K-edge X-ray Absorption Spectroscopy.

**Figure 3.**
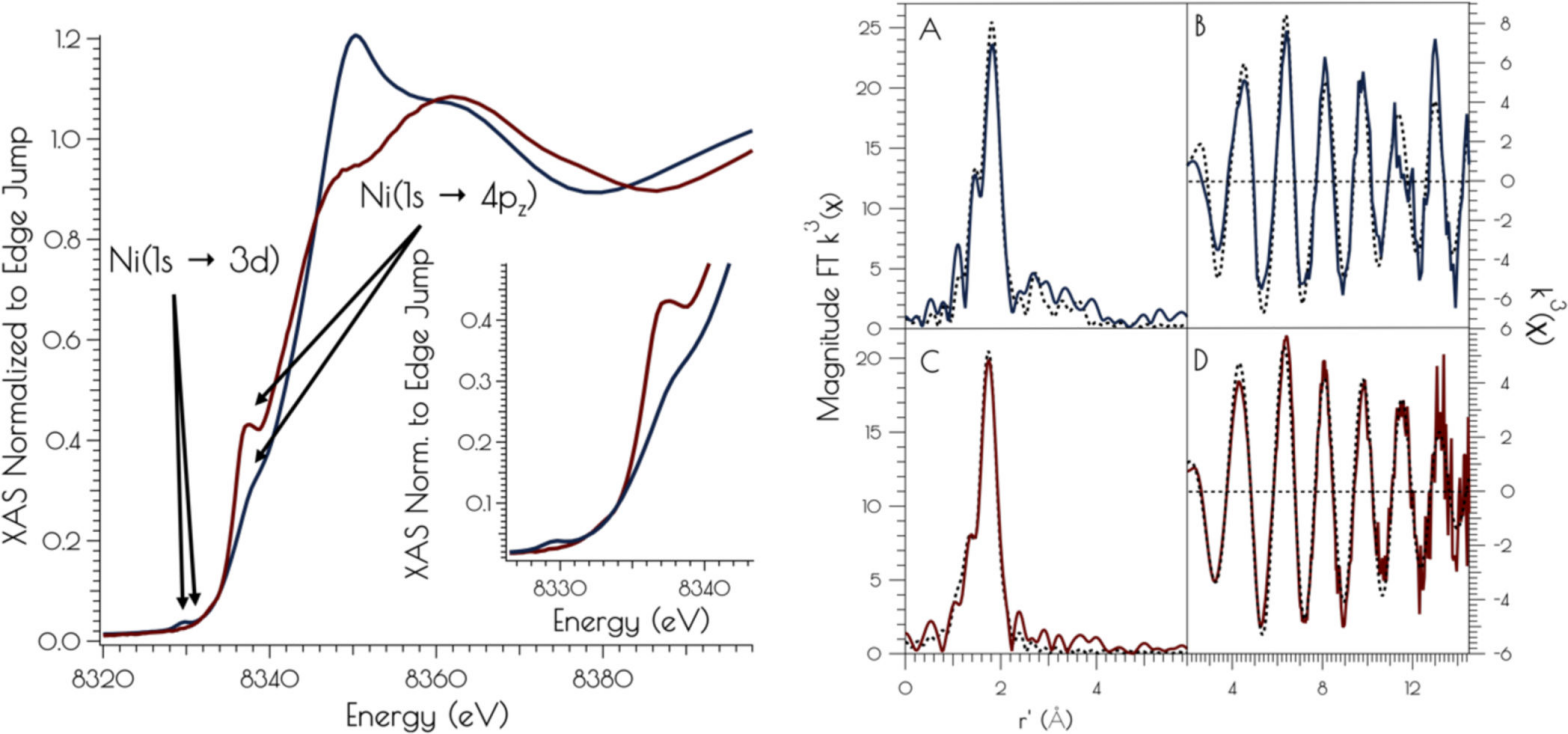
Left: XANES region of the Ni K-edge X-ray absorption spectrum for {Ni_2_^II^(SOD^mds^)} at pH 7.4 (red) and 9.6 (blue). The inset depicts a blow-up of the Ni(1s → 3d) and Ni(1s → 4p_z_) transitions. Right: Magnitude Fourier Transformed k^3^(χ) and unfiltered k^3^(χ) for {Ni_2_^II^(SOD^mds^)} at pH 9.6 (A and B) and 7.4 (C and D). Refinements pH 7.4: a) Ni-S: n = 2; r = 2.1804(14) Å; σ^2^ = 0.0026(2) Å^2^, b) Ni-N: n = 2; r = 1.907(16) Å; σ^2^ = 0.0013(6) Å^2^; σ^2^ = 0.0019(6) Å^2^; E_o_ = 8347.1 eV; ε^2^ = 0.69. Refinements pH 9.6: a) Ni-S: n = 3; r = 2.229(2) Å; σ^2^ = 0.0044(2) Å^2^, b) Ni-N: n = 1; r = 1.889(9) Å; σ^2^ = 0.0013(8) Å^2^, c) Ni-Ni: n = 1; r = 3.25(3) Å; σ^2^ = 0.0061(15) Å^2^; E_o_ = 8346.3 eV; ε^2^ = 1.47.

The nickel K-edge X-ray absorption spectra of {Ni_2_^II^(SOD^mds^)} at pH 7.4 and 9.6 are depicted in Figure 3. The XANES at both pH 7.4 and 9.6 are consistent with Ni^II^ contained in a nomial square planar coordination environment; both display a weak Ni(1s → 3d) transition and a more intense low energy Ni(1s → 4pz) transition.^39^ However, the XANES spectra display notable differences at the two different solution pH values. At a pH of 7.4, the XANES region contains a poorly resolved, weak nominal Ni(1s → 3d) transition at 8333.4(3) eV and a higher energy more intense Ni(1s → 4pz) transition at 8337.3(2) eV that is well separeted from the edge. Raising the pH to 9.6 changes the overall shape of the edge indicating a change in coordination geometry about nickel. In addition, the Ni(1s → 3d) transition becomes more intense and red-shifts to 8331.0(2)eV while the Ni(1s → 4pz) transition becomes less intense, blue-shifts to 8338.2(4) eV, and is now poorly resolved from the edge. These observed changes indicate that a more rigourous square-planar coordination environment about nickel is generated at pH 7.4, which undergoes a distrotion towards nominal *D*_*2d*_ symmetry at pH 9.6 (vide infra).

**Scheme 1.**
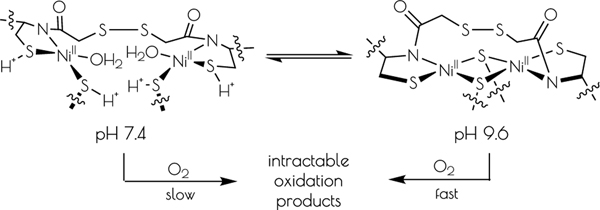
Interconversion of the structures of the nickel-sites of {Ni_2_^II^(SOD^mda^)} at pH 7.4 and 9.6 and subsequent oxidative decomposition upon O_2_ exposure.

At pH 7.4 the EXAFS region of the Ni K-edge X-ray absorption spectrum is best modeled as a four-coordinate Ni-center with two Ni-S scatterers at 2.18 Å and two Ni-N/O scatterer at 1.91 Å. This model is consistent with a nickel center ligated by two cysteinate S atoms, one amidate nitrogen atom and a water ligand, with the N/O scatterers modeled in one shell. At pH 9.6 we see the loss of one N/O scatterer with the subsequent coordination of an addition sulfur ligand; the best fit to the data contained three Ni-S scatterers at 2.23 Å and one Ni-N scatterer at 1.89 Å. In addition, a vector for an outersphere Ni-Ni scatterer could be located at 3.25 Å. These data are consistent with the structural models depicted in Scheme 1; at pH 7.4 the data is consistent with each peptide containing two mononuclear nickel sites that collapses into a binuclear cysteinate bridged Ni_2_ center at high pH, the formation of which is well represented in small molecule Ni-thiolate chemistry.^40–41^

### 2.3. Sulfur K-edge X-ray Absorption Spectroscopy of {Ni_2_^II^(SOD^mds^)}.

**Figure 4.**
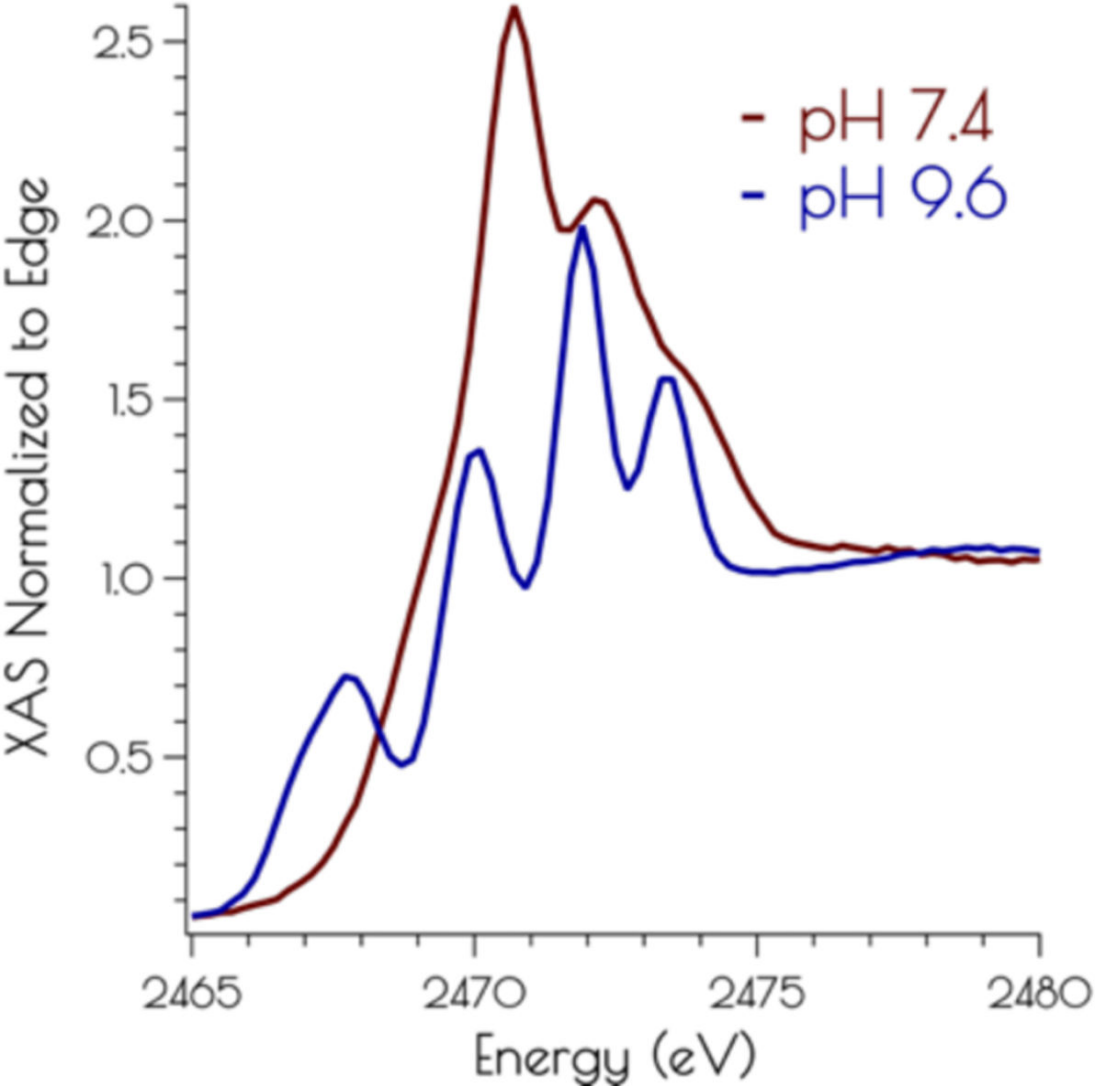
Sulfur K-edge XANES of {Ni_2_^II^(SOD^mds^)} at high 7.4 (red) and 9.6 (blue).

The sulfur K-edge X-ray absorption spectra of {Ni_2_^II^(SOD^mds^)} at pH 7.4 and 9.6 are depicted in Figure 4. At both pH values, there are higher energy edge features that are consistent with a disulfide bond, further demonstrating the presences of a bridging disulfide at both pH 7.4 and 9.6. At high pH there is a pre-edge feature that possess distinct asymmetry, and can be identified as terminal and bridging nickel-cysteinate S(1s → LUMO) transitions.^15^ At pH 7.4 this feature is lost while the edge broadens and gains intensity. This suggests that the S(1s → LUMO) transitions have been blue-shifted into the edge, and is fully consistent with the EXAFS analysis of the metallopeptide at high and low pH; lowering the pH induces the breaking of the bridging Ni-S^Cys^–Ni bonds, generating two mononuclear nickel centers. Furthermore, the sulfur K-edge spectrum at pH 7.4 is consistent with the formation of protonated coordinated Ni-S(H^+^)-Cys moieties at physiological pH; the reversible blue-shift of the S(1s → LUMO) transition upon lowering the pH is a hallmark of the formation of Ni-S(H^+^)-Cys moieties.^15^ This is further validated by time dependent DFT calculations simulating the S K-edge X-ray absorption spectra (vide infra).

### 2.4. DFT Generated Structure for {Ni_2_^II^(SOD^mds^)} and TD-DFT Calculations of Ni and S K-edge XAS Transitions.

**Figure 5.**
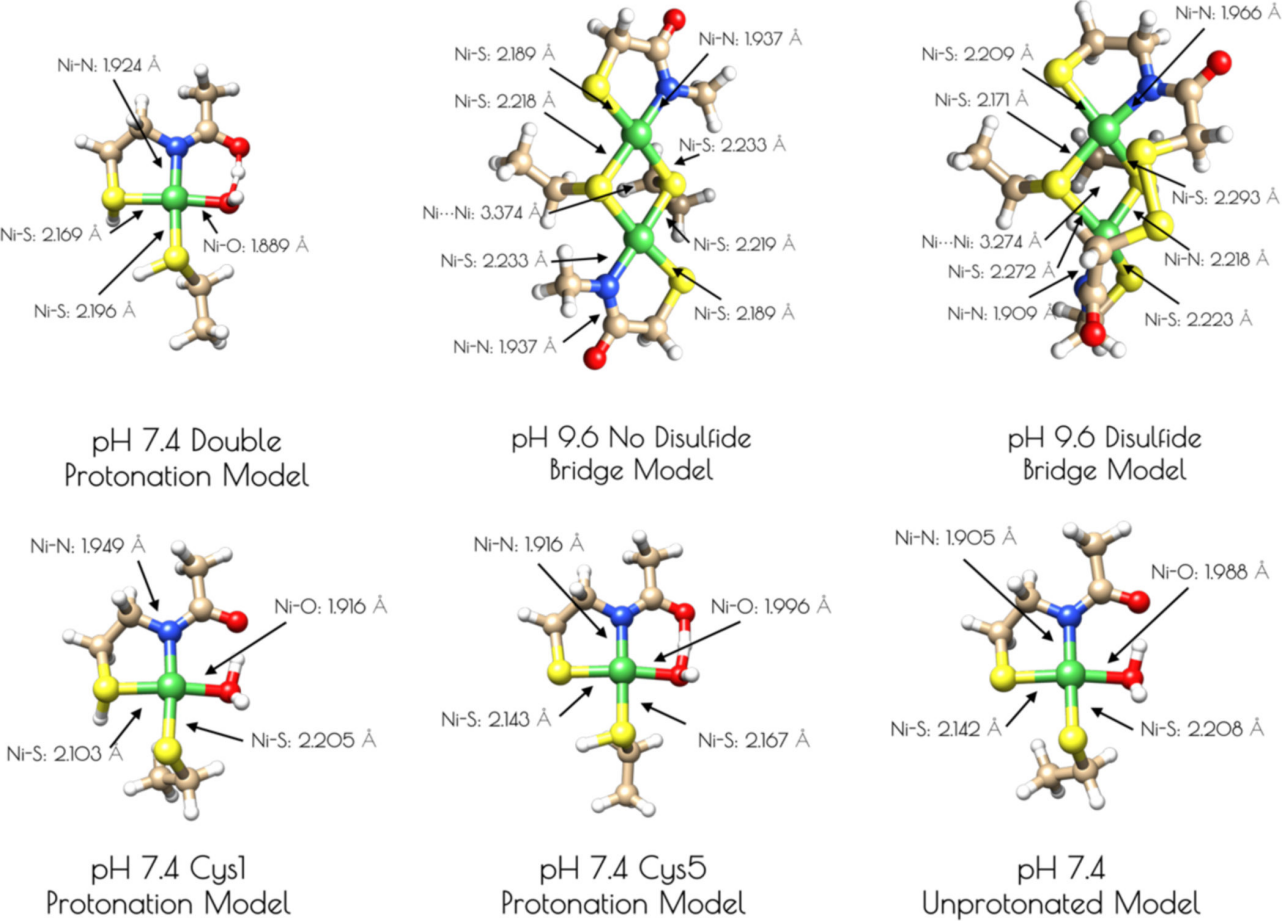
Computationally derived nickel site models of {Ni_2_^II^(SOD^mds^)}. Metric parameters are provided next to the Ni-ligand bonds.

Based on the Ni- and S K-edge XAS data, several computational models of the high and low pH forms of {Ni_2_^II^(SOD^mds^)} were generated (Figure 5). One is a mononuclear nickel site that contains a formal Ni^II^ center in an S_2_NO coordination environment with ligands derived from two thiolate sulfur atoms, an amidate nitrogen atom, and a water ligand. Models of this mononuclear site with variable thiolate sulfur atom protonation were also investigated. As computational models for the pH = 9.6 form of {Ni_2_^II^(SOD^mds^)}, two dinuclear thiolate bridged Ni_2_ models were also generated. One possessed two bridging thiolates, one terminal thiolate and one amidate nitrogen per nickel center. The other dinuclear model possessed an identical Ni_2_S_2_ core, but with a disulfide bond derived from the 2-mercaptoacetate groups bridging the two ligand sets together. Geometry optimizations were performed at the BP86/def2-tzvp level of theory with a dispersion correction; this functional generally yields computationally derived structures for transition metal complexes that are in excellent agreement with experimental data.^42–43^

An examination of the metric parameters derived for the mononuclear Ni-sites (pH 7.4 monomeric model) demonstrates that a model with each thiolate sulfur becoming protonated and a water ligand bound to nickel is most consistent with the experimental parameters derived from EXAFS. The two Ni-S bond lengths are calculated to be 2.169 Å (*trans* to the water ligand) and 2.196 (*trans* to the amidate nitrogen), the Ni-N bond length is calculated to be 1.925 Å and the Ni-O bond length is calculated to be 1.889 Å. These are fully consistent with the EXAFS derived average Ni-S and Ni-N/O bond lengths. The mono- and unprotonated models yield Ni-ligand bond lengths that are less consistent with the EXAFS data than the doubly protonated model. However, when one considers the inherent error in EXAFS derived bond lengths the mono and unprotonated models could still be considered valid structural models for {Ni_2_^II^(SOD^mds^)} based on bond-lengths alone. Nevertheless, the experimental vs theoretical Ni and S K-edge studies strongly suggest the doubly-protonated model is most valid (vide infra).

It was determined that inclusion of the *N*-terminal disulfide bridge is required to reproduce the metric parameters observed in the high pH form of {Ni_2_^II^(SOD^mds^)}. When the disulfide moiety is omitted from the computational model, the geometry about the nickel-sites is nearly planar: the S-Ni-S-Ni dihedral angles are all ~3°. This planar geometry yields a Ni-Ni distance that is 0.1 Å longer than what is observed by EXAFS (3.374 Å). Inclusion of the disulfide bond forces the geometry around the nickel sites to become distorted away from planarity. Furthermore, the two nickel sites are no longer equivalent to one another with one center distorted more toward a tetrahedral-like coordination environment than the other (the above measured dihedral angles are now 33° and 27°). This distortion brings the two Ni-Ni centers closer to one another by 0.1 Å, which reproduces the EXAFS derived Ni-Ni distance nicely (3.274 Å). Furthermore, the average Ni-S and Ni-N bond lengths are more consistent with the EXAFS derived bond lengths. This lends further evidence for the presence of an *N*-terminal disulfide bond.

**Figure 6.**
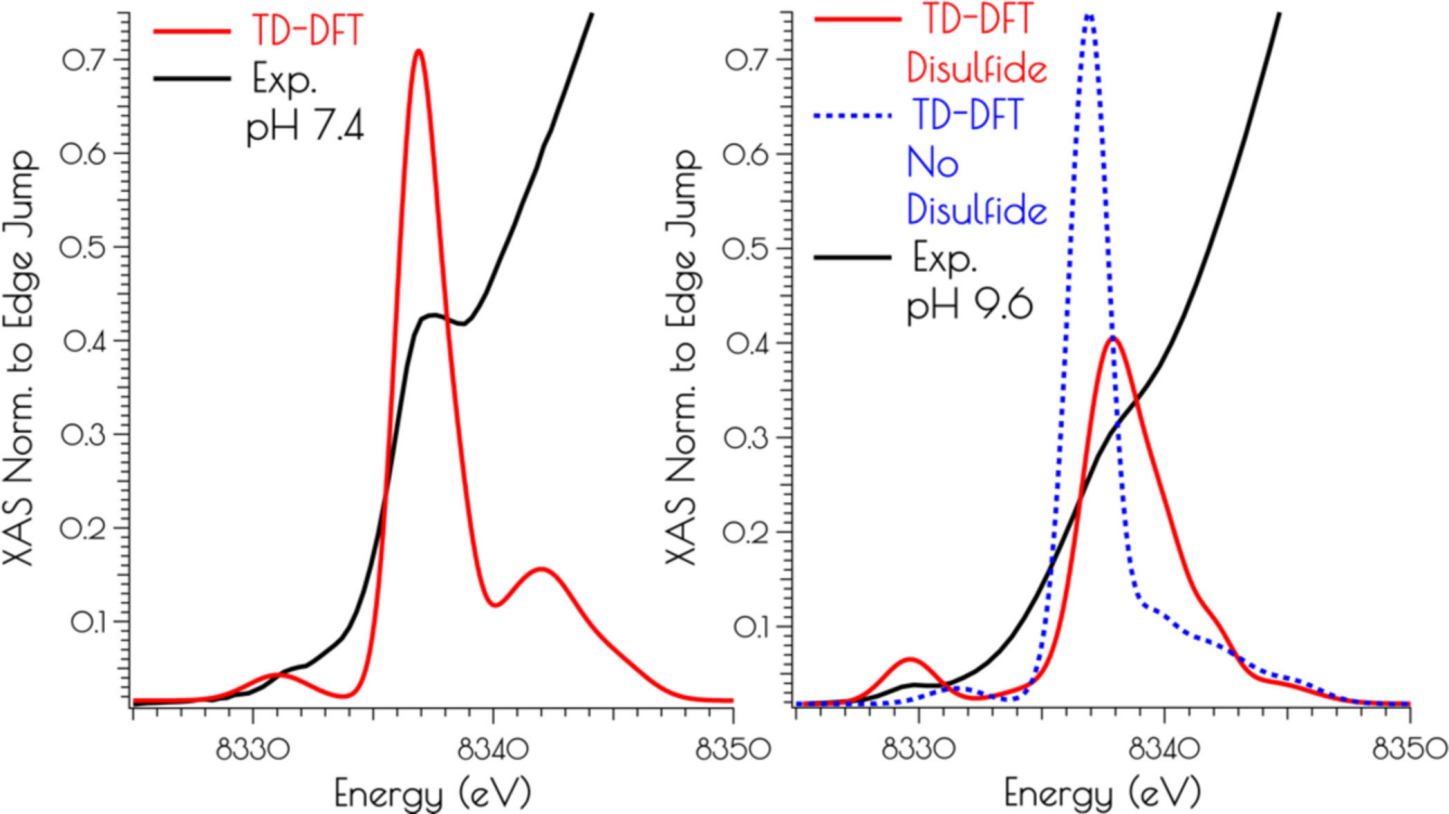
Comparison of the TD-DFT calculated Ni K-edge XANES with the experimental spectra for {Ni_2_(SOD^mds^)} at pH 7.4 (left) and 9.6 (right).

Correlating the experimental Ni K-edge XANES at high and low pH with the time dependent DFT (TD-DFT; PBE0/zora-def2-tzvp(-f)/ZORA) calculated Ni K-edge spectra of the computational models above supports the proposed structures (Figure 6). For the monomeric structures we find that the nearly rigorous square planar coordination environment about Ni^II^ yields Ni(1s → 3d) and Ni(1s → 4p_z_) transitions that correlate well with the experimental data. As might be expected, the pH 9.6 model lacking the disulfide bridge resembles that of the pH 7.4 data, but is inconsistent with the pH 9.6 experimental data. Once the disulfide bridge is included in the computational model the Ni(1s → 3d) transition red-shifts and increases in intensity while the Ni(1s → 4p_z_) transition blue shifts and decreases in intensity. The reason for this is a consequence of 3d/4p mixing. In the *D*_*2d*_ distorted non-centrosymmetric coordination environment, the loss of the pseduo-inversion center allows for the Ni(1s → 3d) transition to acquire 4p character and the Ni(1s → 4p_z_) transition to acquire 3d character. The result is an increase in the intensity of the Ni(1s → 3d) transition by gaining the dipole-allowed Ni(1s → 4p) character while the Ni(1s → 4p_z_) transition losses intensity owing to an increase dipole-forbbiden Ni(1s → 3d) character and a decrease in Ni(1s → 4p) character (Figure 6).

**Figure 7.**
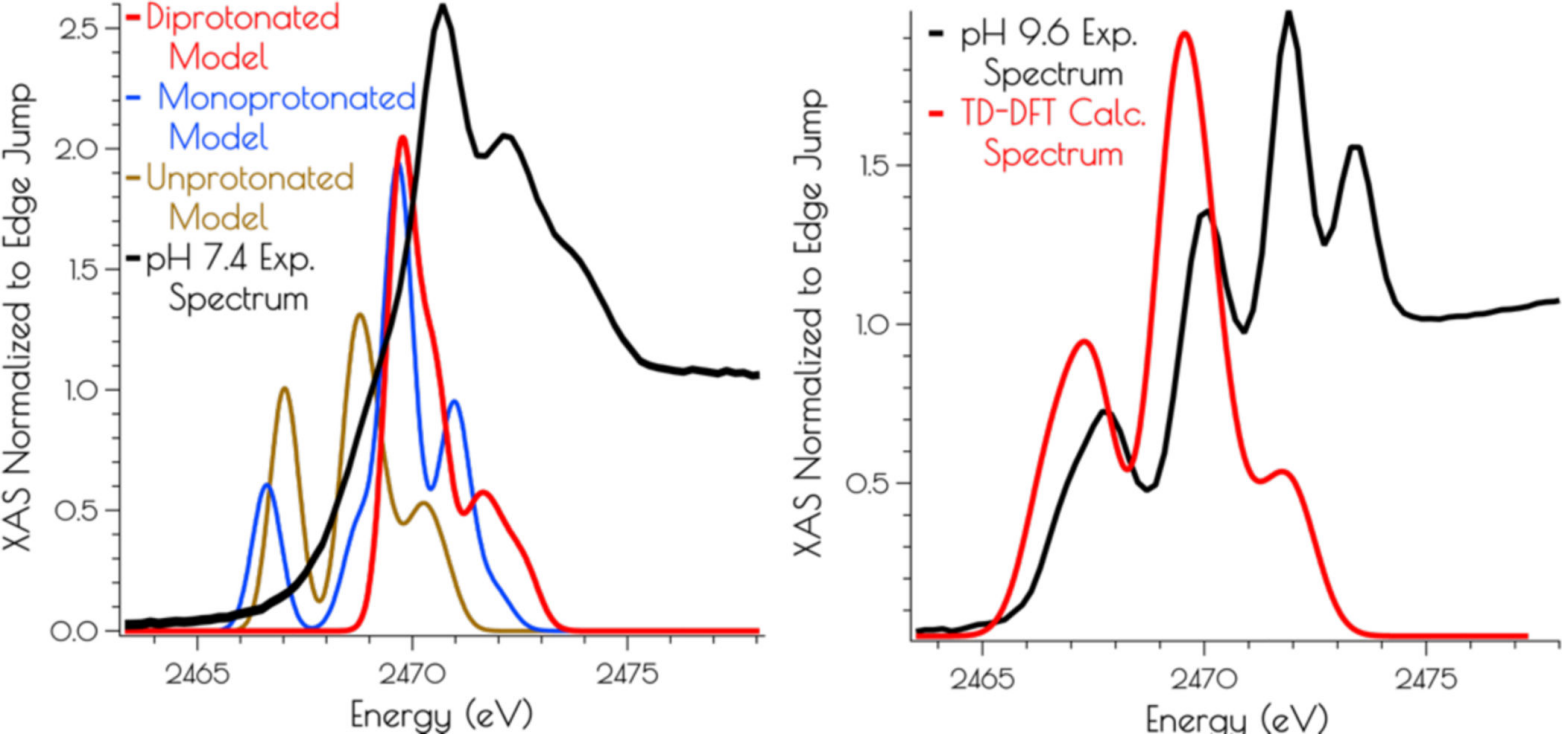
Left: Experimental (black spectrum) pH 7.4 and TD-DFT calculated S K-edge X-ray absorption spectra models of {Ni_2_^II^(SOD^mds^)} (unprotonated model: gold spectrum; monoprotonated model: blue spectrum; doubly-protonated model: red spectrum). Right: Experimental (black spectrum) pH 9.6 and calculated spectrum (disulfide bridged model: red spectrum) of {Ni_2_^II^(SOD^mds^)}.

Calculation of the S K-edge X-ray absorption spectra using time dependent DFT (TD-DFT; PBE0/zora-def2-tzvp(-f)/ZORA) also supports the structural assignments proposed above. The experimental S K-edge X-ray absorption spectrum of {Ni_2_^II^(SOD^mds^)} obtained at pH 9.6 and the TD-DFT calculated S K-edge X-ray absorption spectrum using the disulfide bridged model match well, lending further credence to the validity of the proposed structural model (Figure 7). To determine the likely protonation state of the monomeric pH 7.4 form of {Ni_2_^II^(SOD^mds^)}, the doubly-protonated, monoprotonated, and unprotonated monomeric models outlined above were examined. When one or both of the cysteinate sulfur atom(s) is unprotonated a low energy pre-edge feature corresponding to the nominal S(1s → LUMO) transitions is produced in the calculated spectrum. As would be predicted, this pre-edge feature is weaker for the mono-protonated model relative to the unprotonated model owing to the fact that only one S(1s → LUMO) transition comprises this feature as opposed to two; the S(1s → LUMO) transition for a protonated thiolate sulfur atom will blue shift into the edge. It is therefore only the doubly-protonated model that reproduces the edge feature of the pH 7.4 experimental spectrum of {Ni_2_^II^(SOD^mds^)}. The TD-DFT calculated S K-edge X-ray absorption spectrum for the doubly-protonated model has both S(1s → LUMO) transitions blue shifted by ~3.5 eV relative to the energy of the unprotonated sulfur atom S(1s → LUMO) transition, which reproduce the experimental spectrum well. Thus, we conclude that the pH 7.4 form of {Ni_2_^II^(SOD^mds^)} possesses two protonated cysteinate sulfur atoms coordinated to nickel.

### 2.5. Oxidation of {Ni_2_^II^(SOD^mds^)} at pH 7.4 and 9.6.

**Figure 8.**
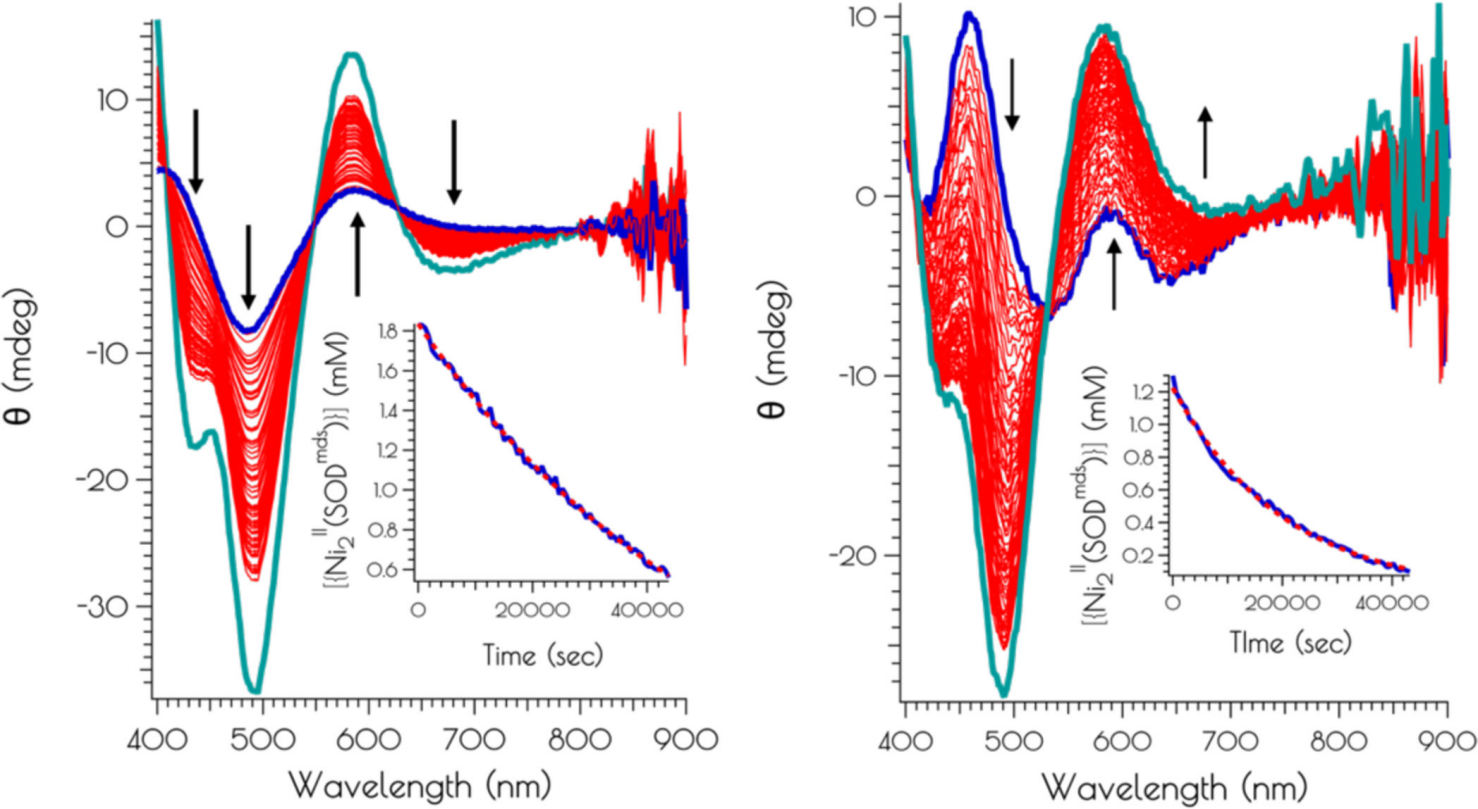
CD spectra following the air oxidation of {Ni_2_^II^(SOD^mds^)} at pH 7.4 (left) and 9.6 (right), with the blue spectra representing the trace at *t* = 0 seconds, the red spectra representing the traces recorded every 600 seconds (10 min) over the course of 12 hours, and the teal spectra represent the CD spectra of the solutions following 24 hours of O_2_ exposure. The insets depict the kinetics traces highlighting the decay of {Ni_2_^II^(SOD^mds^)} (blue trace) and best fit of the kinetic trace to a first order rate law.

{Ni_2_^II^(SOD^mds^)} reacts with O_2_ at both pH 7.4 and 9.6. The kinetics of O_2_ oxidation was followed by CD spectroscopy over the course of 12 hours (Figure 8). It was found that the oxidation kinetics obey a pseudo-first order rate law under constant O_2_ concentration, and proceeds at a faster rate at high vs low pH. Extraction of the second order rate constant for the oxidation reactions demonstrates that the reaction at pH 9.6 proceeds with a rate that is over 3.5-fold faster than at pH 7.4 (*k* = 1.8(3) × 10^−2^ M^−1^ s^−1^ vs 6.5(2) × 10^−2^ M^−1^ s^−1^). Based on an analysis of the products formed during the oxidation reaction, we suspect that following the initial oxidation step there are multiple oxidation pathways leading to different final oxidation products.

There are a number of items to note concerning the oxidation products formed at pH 7.4 and 9.6. First, under both pH conditions, at least one of the final soluble oxidation products contains nickel, and this product is identical by CD under both pH conditions. However, MS data of the solution and solid materials produced by O_2_ initiated oxidation indicates a complex mixture of unidentifiable products. Furthermore, the EPR spectra of the resulting products are silent down to 10 K, indicating the Ni-site is in the formal Ni^2+^ oxidation state. The resulting IR spectra of the produced solutions and solids showed no bands corresponding to S=O stretching frequencies, indicating oxygen atom insertion reactions into the Ni-S moiety does not represent a major oxidation pathway. Instead, it is possible O_2_ is initiating irreversible sulfur based ligand oxidation as has been observed in the work of Darensbourg, for example.^44^ Validating this possibility is the observation the metallopeptide cannot be cleanly oxidized; attempts to chemically oxidize {Ni_2_^II^(SOD^mds^)} at pH 7.4 and 9.6 by a 3% hydrogen peroxide solution, ethanolic I_2_ or MnO_4_^−^ lead to the rapid bleaching of the solution and subsequent formation of unidentifiable tan insoluble aggregates, all of which yielded EPR and IR spectra consistent with the above formed from O_2_ oxidation of the solutions.

A complex mixture of soluble and insoluble nickel containing products is also noted by Ni K-edge X-ray absorption spectroscopy. At both solution pH values, the Ni K-edge XANES no longer contains the prominent Ni(1s → 4p_z_) transition, and is more reminecent of a six coordinate Ni^2+^ species. Because of the low signal to noise at high *k*, the EXAFS regions could only be simulated to *k* = 11 Å^−1^ The EXAFS region of the decomposition product obtained from air oxidation at pH 7.4 was best modeled as containing 1.4 Ni-S interactions (r = 2.22 Å) and 4.8 N/O interactions (r = 1.97 Å). The EXAFS region of the air oxidation product generated at pH 9.6 was best modeled with 0.6 Ni-S interactions (r = 2.24 Å), 4.3 N/O interaction (r = 1.94 Å), and 2.1 Ni-Ni interactions (r = 3.25 Å). In both cases, the resulting fitting statistics are poor with ε^2^ values greater than 3. As this represents a mixture of soluble and insoluble compounds in a number of coordination environments, formulating likely structures about the nickel center(s) is not warrented based on the available data.

**Figure 9.**
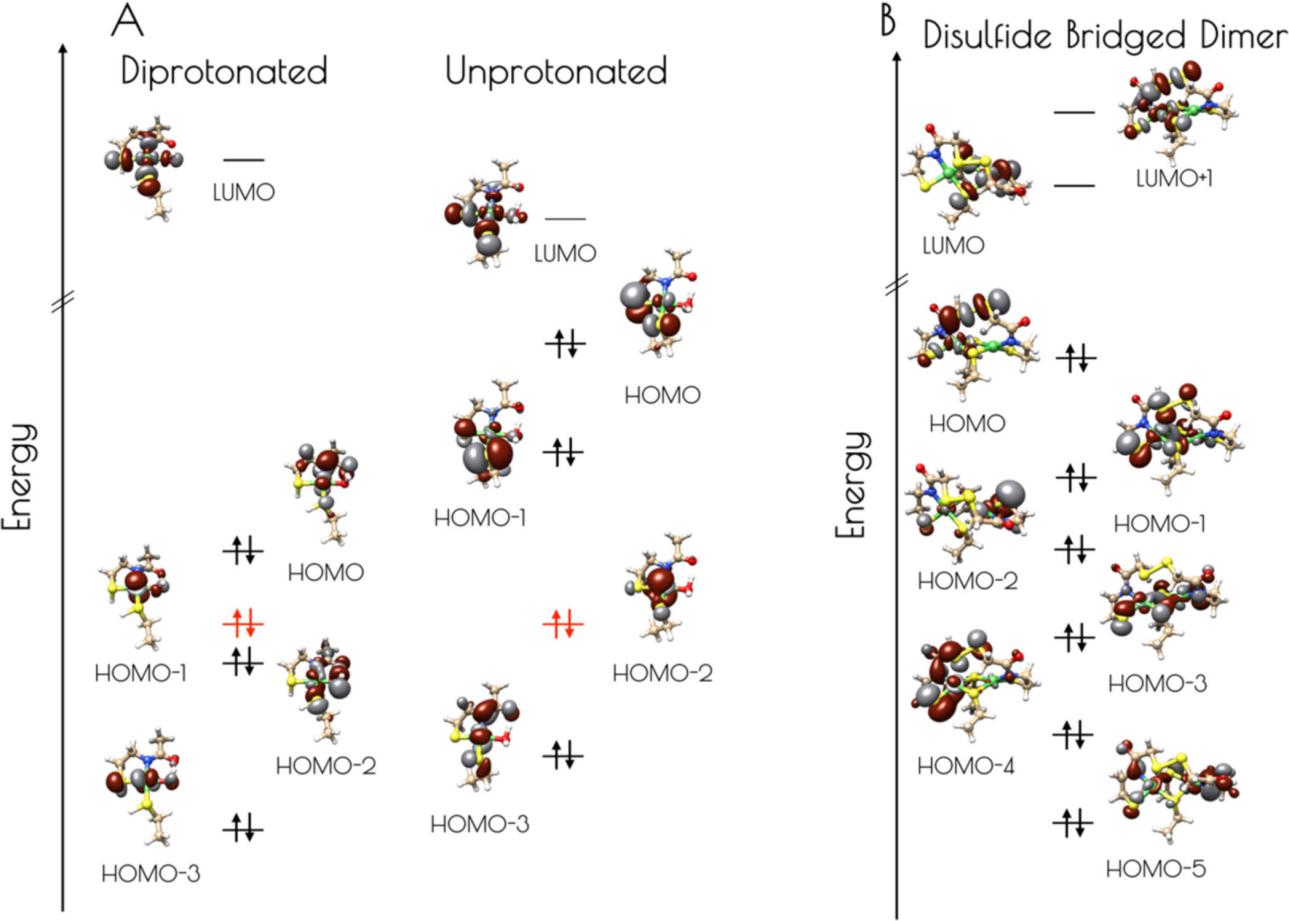
A) Isosurface plots (0.03 a.u.) of the LUMO through HOMO-3 of the doubly-protonated (left) and unprotonated (right) computational models of the pH 7.4 form of the nickel-site of {Ni_2_(SOD^mds^)}. The energies were normalized to the non-bonding Ni(3d_z2_) orbital, highlighted in red. B) Isosurface plots (0.03 a.u.) of the LUMO+1 through HOMO-5 of the disulfide bridged dinuclear {Ni_2_(SOD^mds^)} computational model.

Electronic structure calculations suggest the reason for the increased stability of {Ni_2_(SOD^mds^)} at pH 7.4 vs 9.6 results from the deactivation of the high-lying sulfur dominated Ni(3dπ)-S(3pπ) anti-bonding orbital upon protonation (Figure 9, Tables 1 and 2). For the monomeric species, the HOMO is identified as a nickel dominated Ni(3dπ)-N(2pπ) anti-bonding orbital. This is destabilized by 0.29 eV relative to the essentially non-bonding Ni(3d_z2_) orbital (HOMO-1). The HOMO-2 is a water O(2p) dominated O(2pπ)-Ni(3dπ) antibonding orbital followed by the Ni(3d_xz_) dominated HOMO-3. Thus, none of the frontier MOs (FMOs) possess significant S(3p) character, rendering the sulfur atoms reasonably unreactive towards oxidative damage by O_2_. In contrast, deprotonation of the sulfur atoms of the mononuclear {Ni_2_^II^(SOD^mds^)} nickel-site dramatically alters the electronic structure of the complex. Electronic structure calculations reveal that the HOMO and HOMO-1 are significantly activated relative to the essentially non-bond Ni(3d_z2_) HOMO-2 by 0.73 and 0.42 eV, respectively. Furthermore, these two orbitals are S(3p) dominated S(3pπ)-Ni(3dπ) anti-bonding orbitals. Therefore, if the deprotonated monomeric form of {Ni_2_(SOD^mds^)} could be generated, we would predict it would be highly sensitive to O_2_ damage owing to the activated S(3p) dominated HOMO. Given the slow rate of O_2_ oxidation of {Ni_2_(SOD^mds^)} at pH = 7.4, it is likely that deprotonation of the Ni-S(H^+^)-Cys moieties are required for oxidative damage; as protonation is an equilibrium process, there will always be a small concentration of O_2_ reactive unprotonated Ni-S-Cys bonds in solution. The disulfide bridged computational model was found to possess highly covalent Ni-ligand bonds. The HOMO, although containing less S(3p) than the HOMO of the unprotonated mononuclear computational model, still contains a significant amount of S(3p) character, and would therefore be predicted to be more susceptible to O_2_ damage than the doubly-protonated mononuclear nickel-site. This is observed experimentally.

## 3. Discussion

In this study we have demonstrated the reversible formation of a dinuclear Ni_2_^II^ site within a peptide in response to pH. Dinuclear Ni_2_^II^ sites are not observed in other monomeric SOD metallopeptide-based mimics while the disulfide linked dimeric metallopeptide facilitates formation of a dinuclear Ni_2_ center. We speculate that dimer formation in {Ni_2_(SOD^mds^)} is facilitated by the close proximity of the two metal centers, which was enforced through a disulfide bond linkage near the individual nickel centers.

**Figure 10.**
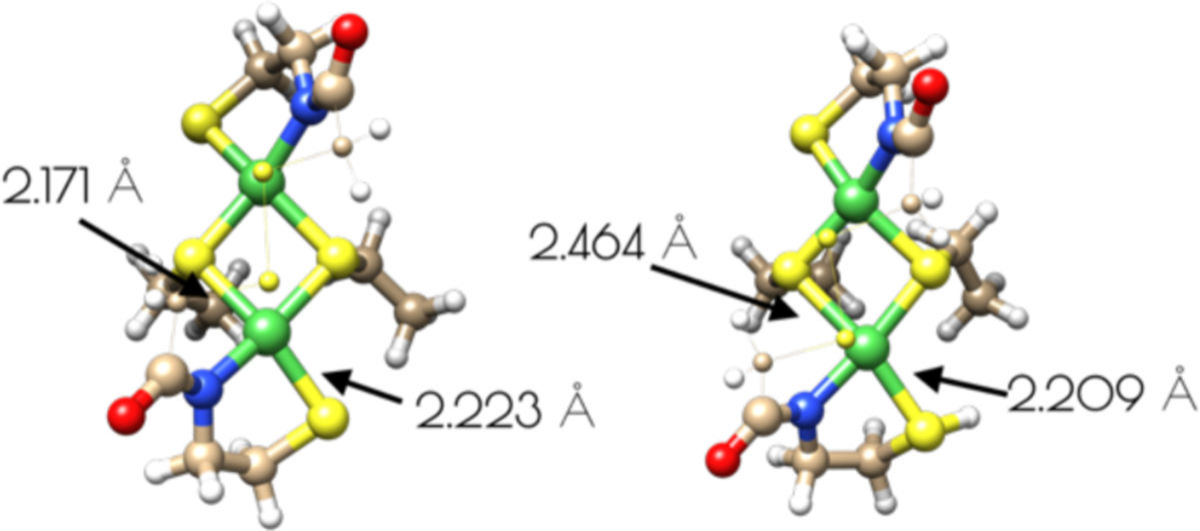
Structures of binuclear nickel-site models of {Ni_2_^II^(SOD^mds^)} and {Ni_2_^II^(SOD^mds^-S(H^+^)C1)}. The disulfide bridge and methylene groups have been represented as small spheres and wires for clarifty.

The driving force for conversion of the dinuclear Ni_2_^II^ site into two monomeric Ni^II^ sites within {Ni_2_(SOD^mds^)} likely involves protonation of a terminal cysteinate sulfur atom. The nucleophilic HOMO of the dinuclear Ni_2_^II^ site possesses S(3p) character corresponding to the terminal thiolate sulfur coordinated to the more distorted nickel center, making it the likely protonation site. Protonation of that sulfur atom shortens the Ni-S(H^+^)-Cys bond length relative to the unprotonated model with a concomitant increase in the bridging Ni-S bond length *trans* to the protonation site (Figure 10). Association of water to the nickel center followed by breaking of the weakened Ni-S bond would lead to the eventual formation of two mononuclear Ni^II^ centers.

This study also gives insight into an additional mechanism of protection of Ni-thiolate bonds from oxidative damage against reactive oxygen species (ROSs). Nickel thiolates are susceptible to oxidative damage by O_2_ and H_2_O_2_,^45–46^ yet the NiSOD active-site is robust against oxidative damage effected by such species. Possible explanations for protection of the NiSOD active-site against Ni-S oxidative damage by ROSs have been proposed, including: electronic fine tuning of the Ni-S moiety via the mixed amine/amide coordination environment^47–48^ and a fast rate of the O_2_^−^ disproportionation reaction relative to the rate of oxidation of the coordinate cysteinate sulfur atoms.^46, 49–50^ In this study we have shown that oxidation of the Ni-S-Cys bond by O2 is slow for {Ni_2_(SOD^mds^)} at pH = 7.4, with a half-life of nearly 7 hours under the reaction conditions investigated (ambient O_2_ concentration, 1.0 mM {Ni_2_(SOD^mds^)}). This is the result of both the deactivation and a significant reduction in S(3p) character to the nucleophilic FMOs upon protonation; the FMOs of the doubly-protonated monomeric {Ni_2_(SOD^mds^)} computational model possesses little S(3p) character, while the HOMO and HOMO-1 of the unprotonated {Ni_2_(SOD^mds^)} computational model are both energetically activated and possess a large degree of S(3p) character. Thus, protonation will inherently protect the thiolate sulfur atoms against ROS induced oxidative damage. The dinuclear nickel site of {Ni_2_(SOD^mds^)} produced at pH = 9.6 was found to undergo oxidation at an increased rate relative to the mononuclear pH = 7.4 form. This is to be expected as the degree of terminal cysteinate S(3p) character to the more covalent anti-bonding FMOs has increased relative to the mononuclear pH = 7.4 doubly-protonated Ni form, rendering it more susceptible to oxidative damage.

This study has also pointed to an additional role for the protonation of coordinated cysteinate sulfur atoms at metalloenzyme active-sites – poising the centers for reactivity via electronic fine tuning. As demonstrated above, the FMOs of the doubly-protonated mononuclear {Ni_2_(SOD^mds^)} are biased to the nickel-site, while those of the unprotonated mononuclear {Ni_2_(SOD^mds^)} computational model are biased to the thiolate sulfur atoms. Thus, one would expect that upon thiolate sulfur atom protonation reactivity would be shifted from the thiolate sulfur atoms to the nickel-site. For example, one could consider the active-site of [NiFe]H_2_ase, which has been shown to possess a terminal protonated cysteinate residue ligated to a *D*_*2d*_ distorted nickel center. It has been proposed that a key intermediate in the reactivity of [NiFe]H_2_ase is a formal Ni^III^-H species. Protonation of the cysteinate sulfur atom would bias the reactivity towards nickel, making the nickel-center the site susceptible to subsequent protonation events. Thus, one may envision a dual role for the Ni-S(H^+^)-Cys moiety in [NiFe]H_2_ase – gating of reactivity and proton donation to the hydride ligand.

## 4. Conclusion

A disulfide bridged metallopeptide has been prepared inspired by the metalloenzyme NiSOD. This metallopeptide contains two nickel centers in close proximity owing to a disulfide bridge between two peptide monomers. In response to pH the mononuclear nickel-sites found at pH 7.4, which contain Ni^II^ in an S_2_NO coordination motif reversibly form a dinuclear cysteinate Ni_2_^II^ center at elevated pH (pH 9.6). The driving force for the interconversion of the dinuclear nickel center to two mononuclear nickel sites is proposed to be cysteinate S-atom protonation, which results in two coordinated protonated Cys S atoms at lower pH. It was shown that these Ni-S(H^+^)-Cys moieties reduce the O_2_ initiated oxidative damage of the nickel-site, likely through the modulation of the electronic structure of the Ni-center rendering the S-atoms less nucleophilic upon protonation. This may have relevance in biological Ni systems, offering the Ni-S-Cys moiety protection against oxidative damage upon Ni-S(H^+^)-Cys formation. Furthermore, the modulation of the electronic structure of the Ni-site upon Ni-S(H^+^)-Cys formation suggests that reversible cysteinate sulfur atom protonation may be involved in the gating of biological reactivity at such metal-centers.

## 5. Materials and Methods

### 5.1. General Considerations.

All manipulations were performed under an N_2_/H_2_ (97:3) atmosphere in a COY anaerobic chamber. Fmoc/O^*t*^Bu protected amino acids and resins were obtained from Advanced Chemtech (Louisville, KY). All other reagents obtained from commercial suppliers were of the highest purity available, and used as received. Analytical and semi-preparative reverse-phase HPLC were performed using Waters X-Bridge C-18 analytical (4.6 × 150 mm; 5 μm) and semi-preparative (30 × 150 mm; 5 μm) columns on a Waters Deltaprep 600 equipped with a photodiode array detector (detection wavelength set to 254 nm). Mass specrtrometry was performed on either a Bruker Microflex MALDI-TOF mass spectrometer, a ThermoFinnegan LCQ Deca XP ESI-MS, or a Waters Micromass 20 ESI-MS operating in positive ion mode. NMR spectra were obtained on a 400 MHz Varian VNMRS NMR spectrometer. All chemical shifts (*δ*) are referenced to the residual protio solvent peak. Electronic absorption spectra were obtained on either a JASCO J-1500, CARY 50 or CARY 5000 UV-vis-NIR spectrometer. Circular dichroism spectra were obtained on a JASCO J-1500 spectropolarimeter. The simultaneous deconvolutions of the CD and electronic absorption spectra were performed using an in-house-written procedure for Igor Pro version 6 and 8 (Wavemetrics; Lake Oswego, OR). Infrared spectra were collected using a Thermo Nicolet 6700 FTIR spectrometer with a diamond crystal ATR. X-band EPR spectra were obtained on a Bruker EMXPlus EPR spectrometer equipped with a closed-cycle He cryostat.

### 5.2. Preparation of S-triphenylmethyl-thioglycolic acid.

S-triphenylmethyl-thioglycolic acid (T^A^-trityl) was prepared by a modification of the procedure on Martinage et al.^51^ Briefly, 1.5 g (5.77 mmol) of triphenylmethanol was added to a 25 mL TFA solution of thioglycolic acid (400 μL, 5.77 mmol) and stirred for 5 h under argon at room temperature. The TFA was removed under vacuum, and the resulting orange solid was washed three times with toluene followed by three times with hexanes resulting in a white solid, which was analytically pure (1.46 g, 81% yield). ^1^H NMR (CDCl_3_, 400 MHz): *δ* 7.30 (m, 15H), 3.04 (s, 2H).

### 5.3. Preparation of {Ni_2_(SOD^mds^)}.

The peptide SOD^mds^ (T^a^CDLP-CGVYD-PA) was prepared on an AAPTec Focus XC-2RV peptide synthesizer or by manual solid-phase peptide synthesis using HBTU/HOBt coupling strategies on a 0.12 mmole scale with alanine loaded Wang resin using a five-fold excess of activated protected amino acid. Following the coupling trityl protected Cys1 and removal of the Fmoc group, T^A^-trityl was coupled to the *N*-terminus using standard HBTU/HOBt coupling strategies. Global peptide deprotection and peptide cleavage from the resin was performed under N_2_ using a cleavage cocktail comprised of 84.5:5:5:5:2.5 TFA:phenol:water:thioanisole:EDT over the course of 12 hours. Following removal of the cleavage solution by vacuum on a Schlenk line, the resulting glassy product was washed four times with cold freshly distilled diethyl ether. HPLC and MALDI-TOF studies of the crude peptide mixture demonstrated the complete formation of the disulfide bridged dimer. The resulting crude peptide was subsequently purified by preparative HPLC (9:1 water:acetonitrile −4:6 water:acetonitrile over the course of 30 min; rt = 14.6 min) resulting in the pure disulfide bridged dimer (m1S_3_)_2_ (14% yield). MALDI-TOF MS: [SOD^mda^/Na]^+^ exp: 2473.3 m/z; calcd: 2473.9 m/z.

Solutions of SOD^mds^ in 50 mM NEM buffer (pH 7.4 or 9.5) were prepared and 2.0 equiv of NiCl_2_ (added from a pH 7.0 50 mM stock solution) per peptide were then added to solutions of the (m1S_3_)_2_. The number of free thiol groups per peptide was verified using an Ellman’s assay compared to the peptide concentration as determined by the combined absorbance of the Y residue and disulfide moiety (combined ε = 1,525 M^−1^ cm^−1^ at 278 nm).^38^ ESI-MS data were obtained by injecting an air-free solutions of the pH 7.4 or 8.6 metallopeptide into the mass spectrometer using an air-tight syringe (ESI-MS: pH 9.6 [{Ni_2_(SOD^mds^)}/Na]^+^ exp: 2589.1; calcd. 2589.8; ESI-MS: pH 7.4 [{Ni_2_(SOD^mds^)}/Na/H_2_O]^+^ exp: 2607.6; calcd. 2607.8).

### 5.4. Determination of Ni-S(H^+^)-Cys pK^a^.

Solutions of {Ni_2_(SOD^mds^)} were formed in 50 mM NEM buffer at a pH of 6.5. To these, aliquots of NaOH or HCl (0.5 M) were then added to the solution and the pH measured using an Orion^®^ micro-pH electrode and the electronic absorption measured following each addition. The resulting pH titration curve was constructed by monitoring the change in absorbance at λ = 320 nm, where the largest difference in absorbance between the two species is observed.

### 5.5. Kinetics of the Air Oxidation of {Ni_2_^II^(m1S_3_)_2_}.

The circular dichroism (CD) spectrum of anaerobically prepared solutions of 1.0 mM {Ni_2_(SOD^mds^)} at pH 7.4 or 9.5 (50 mM NEM buffer), and then air was bubbled through the metallopeptide solutions for five min. A CD spectrum of these solutions was subsequently taken every 10 min for 12 hours with the sample continuously exposed to air. The oxidation kinetics was modeled using pseudo-first-order reaction kinetics using *KinTek Explorer v 5.2*. Second order rate constants are reported per nickel site – one at pH = 9.6 and two at pH = 7.4. From the deconvoluted spectra, kinetic traces are reported in the decay of the {Ni_2_(SOD^mds^)} starting materials.

### 5.6. Nickel K-edge X-ray Absorption Spectroscopy.

Nickel K-edge X-ray absorption spectroscopic data were collected on the HXMA beamline (wiggler insertion device operating at 1.5 T) at the Canadian Light Source (Saskatoon, SA, Canada). Solutions of {Ni_2_(SOD^mds^)} (1.0 mM in 1:1 50 mM NEM buffer:glycerol at a pH of 7.4 or 9.5) were injected between Kapton tape windows in aluminum sample holders and quickly frozen in liquid nitrogen. Data were collected at 20 K with sample temperatures maintained using an Oxford liquid He flow cryostat. Light was monochromatized using a Si(220) double crystal monochromator, which was detuned 50% for harmonic rejection, and focused using a Rh mirror. Spectra were obtained in fluorescence mode using a 32 element solid-state Ge detector on both lines with a 3 micron cobalt filter placed between the sample and detector, and spectra were calibrated against the first inflection point of Ni-foil (8333 eV), which was simultaneously recorded with the metallopeptide data. Data were collected in 10 eV steps from 8133 – 8313 eV (1 second integration time per point), 0.3 eV steps from 8313 – 8363 eV (3 second integration time per point), 2 eV steps from 8363 – 8633 eV (5 second integration time per point), and 5 eV steps from 8633 eV – 16 k (5 second integration time per point). Total fluorescence counts were maintained under 30 kHz, and a deadtime correction yielded no appreciable change to the data. The reported spectra represent the averaged spectra from 5 individual data sets. Prior to data averaging each spectrum and detector channel was individually inspected for data quality. Data were subsequently processed and analyzed as previously reported using *EXAFS123* and *FEFF 9.4*.^18^

### 5.7. Sulfur K-edge X-ray Absorption Spectroscopy.

Solutions of {Ni_2_(SOD^mds^)} were prepared at a pH of either 7.4 or 9.6 (~1 mM in 50 mM NEM buffer) and injected into Lucite sample holders with polypropylene windows. Data were obtained at room temperature (~20 °C) on beamline X-19a at the NSLS (Upton, NY) in a He purged sample chamber using a passivated implanted planar silicon (PIPS) detector. The photon energies were calibrated against the first inflection point of S_8_ recorded before and after each sample; it was found that there was no detectable monochromator drift throughout the data collection. Data were obtained in 5 eV steps in the pre-edge region, 0.1 eV steps in the edge region, 1 eV steps in the near edge region, and 5 eV steps in the far edge region. The reported data represents the average of five individual scans. Following data averaging and a baseline was applied to each spectrum by fitting the pre-edge region to a polynomial function. This baseline was then subtracted from the whole spectrum. The region above the edge jump was then fit to a two-knot cubic spline, and the data normalized to the edge height.

### 5.8. Electronic Structure Calculation.

Electronic structure calculations were performed using ORCA v 4.1.0.^52^ Unless otherwise stated, all calculations employed Ahlrichs’ def2-tzvp basis set^53^ on all atoms and the atom pairwise dispersion correction with Becke-Johnson damping to account for dispersive interactions.^54–55^ ORCA VeryTightSCF convergence criteria were used for the SCF cycles, with program defaults used for all other convergence criteria and settings. Geometry optimizations were performed at the BP86 level, and used the RI approximation and def2-tzvp/c auxiliary basis set.^56^ Single point calculations were performed at the PBE0 level and used the RIJCOSX approximation and def2-tzvp/j auxiliary basis set.^57^

Nickel and sulfur K-edge X-ray absorption spectra were simulated using TD-DFT calculations (PBE0/def2-tzvp(-f) and the ZORA relativistic approximation) examining the first 25 transitions originating from each sulfur atom or the nickel atom. A Gaussian function to each transition (FWHM = 0.75 eV for S K-edge calculations and 1.2 eV for Ni K-edge calculations) followed by summing the individual transitions was used to produce the calculated spectra. A +36.7 eV (sulfur K-edge) or +171.3 eV (nickel K-edge) energy correction was applied to each transition. Atomic orbital population analyses were performed using a Löwdin population analysis. Isosurface plots were generated using Chimera v 1.13.1.^58^

## Supplementary Material

SI

Table S2

Table S3

Table S1

Table S4

Table S5

Table S6

Table S7

Table S8

Table S9

Fig S1

FigS2

## Figures and Tables

**Table 1.** Ni(3d), S(3p), N(2p), and water O(2p) Löwdin MO population analysis (%AO to MO) and energies (eV) relative to the Ni(3d_z2_) orbital for the LUMO through HOMO-3 of the computational models for doubly-protonated and unprotonated monomeric {Ni_2_(SOD^mds^)} computational models. Orbital compositions for the doubly-protonated model are given above the unprotonated model for each AO.

	LUMO	HOMO	HOMO-1	HOMO-2	HOMO-3
Ni doubly-protonated unprotonated	61.4 51.7	48.9 29.9	76.8 16.2	29.9 76.3	80.5 61.7
S^1^ doubly-protonated unprotonated	6.0 11.5	1.6 9.9	2.4 55.7	7.4 5.6	0.0 0.3
S^2^ doubly-protonated unprotonated	3.9 11.6	0.2 46.1	1.1 10.1	0.1 6.5	6.4 4.9
N doubly-protonated unprotonated	3.9 2.1	28.5 0.2	2.7 10.0	3.3 11.0	0.0 5.3
O doubly-protonated unprotonated	7.4 3.6	0.2 0.6	6.7 3.0	41.0 0.9	3.7 4.8
E doubly-protonated unprotonated	5.36 5.12	0.29 0.73	0.00 0.42	–0.07 0.00	–0.49 –0.22

1 *trans* to amidate nitrogen

2 *trans* to water oxygen.

**Table 2.** Ni(3d), S(3p), and N(2p) Löwdin MO population analysis (%AO to MO) and energies (E, eV) relative to the HOMO for the LUMO+1 through HOMO-5 of the computational model for the disulfide bridged dinuclear {Ni_2_(SOD^mds^)} nickel site computational model. Orbital compositions for the doubly-protonated model are given above the unprotonated model for each AO.

	LUMO+1	LUMO	HOMO	HOMO-1	HOMO-2	HOMO-3	HOMO-4	HOMO-5
Ni^1^	0.3	21.1	28.2	4.8	11.8	20.3	7.5	18.5
Ni^2^	50.6	0.4	4.3	23.3	11.7	1.7	24.6	38.4
S^3^	0.4	16.4	10.4	1.5	1.2	10.2	0.1	0.4
S^4^	0.4	17.1	5.2	0.2	0.6	88	0.8	7.3
S^5^	6.1	2.0	4.2	2.0	9.5	0.6	1.2	1.1
S^6^	8.7	2.4	9.5	1.4	8.9	0.4	6.0	1.4
S^7^	0.1	5.1	21.9	4.6	15.6	30.0	6.1	5.1
S^8^	10.1	0.0	3.2	49.2	15.2	0.4	2.3	4.3
N^9^	0.2	1.6	0.4	0.2	1.7	6.1	5.7	5.5
N^10^	3.0	0.0	0.2	0.9	5.0	0.8	14.9	3.8
E	3.76	3.62	0.00	–0.30	–0.54	–0.69	–0.72	–1.07

1 more distorted nickel site

2 less distorted nickel site

3 disulfide sulfur over Ni^1^

4 disulfide sulfur over Ni^2^

5 bridging thiolate sulfur

6 bridging thiolate sulfur

7 terminal thiolate sulfur ligated to Ni^1^

8 terminal thiolate ligated to Ni^2^

9 amidate nitrogen ligated to Ni^1^

10 amidate nitrogen ligated to Ni^2^.

## References

[1] Can M; Armstrong FA; Ragsdale SW, Structure, function, and mechanism of the nickel metalloenzymes, CO dehydrogenase, and acetyl-CoA synthase. Chem. Rev 2014, 114 (8), 4149–4174. 24521136 10.1021/cr400461p PMC4002135

[2] Maroney MJ; Ciurli S, Nonredox nickel enzymes. Chem. Rev. 2014, 114 (8), 4206–4228. 24369791 10.1021/cr4004488 PMC5675112

[3] Ragsdale SW, Biochemistry of methyl-coenzyme M reductase: the nickel metalloenzyme that catalyzes the final step in synthesis and the first step in anaerobic oxidation of the greenhouse gas methane. Met. Ions Life Sci. 2014, 14 (Metal-Driven Biogeochemistry of Gaseous Compounds in the Environment), 125–145. 25416393 10.1007/978-94-017-9269-1_6

[4] Ragsdale SW, Nickel biochemistry. Curr. Opin. Chem. Biol. 1998, 2 (2), 208–215. 9667931 10.1016/s1367-5931(98)80062-8

[5] Ragsdale SW, Nickel-based enzyme systems. J. Biol. Chem. 2009, 284 (28), 18571–18575. 19363030 10.1074/jbc.R900020200 PMC2707248

[6] Higgins KA; Carr CE; Maroney MJ, Specific metal recognition in nickel trafficking. Biochemistry 2012, 51 (40), 7816–7832. 22970729 10.1021/bi300981m PMC3502001

[7] Chung KCC; Cao L; Dias AV; Pickering IJ; George GN; Zamble DB, A High-Affinity Metal-Binding Peptide from Escherichia coli HypB. J. Am. Chem. Soc. 2008, 130 (43), 14056–14057. 18834129 10.1021/ja8055003

[8] Dias AV; Mulvihill CM; Leach MR; Pickering IJ; George GN; Zamble DB, Structural and Biological Analysis of the Metal Sites of Escherichia coli Hydrogenase Accessory Protein HypB. Biochemistry 2008, 47 (46), 11981–11991. 18942856 10.1021/bi801337x

[9] Douglas CD; Ngu TT; Kaluarachchi H; Zamble DB, Metal Transfer within the Escherichia coli HypB-HypA Complex of Hydrogenase Accessory Proteins. Biochemistry 2013, 52 (35), 6030–6039. 23899293 10.1021/bi400812r PMC4457517

[10] Lacasse MJ; Douglas CD; Zamble DB, Mechanism of Selective Nickel Transfer from HypB to HypA, Escherichia coli [NiFe]-Hydrogenase Accessory Proteins. Biochemistry 2016, 55 (49), 6821–6831. 27951644 10.1021/acs.biochem.6b00706

[11] Schreiter ER; Sintchak MD; Guo Y; Chivers PT; Sauer RT; Drennan CL, Crystal structure of the nickel-responsive transcription factor NikR. Nature Structural & Molecular Biology 2003, 10, 794–799. 10.1038/nsb985 12970756

[12] Clegg W; Henderson RA, Kinetic Evidence for Intramolecular Proton Transfer Between Nickel and Coordinated Thiolate. Inorg. Chem. 2002, 41 (5), 1128–1135. 11874347 10.1021/ic0104306

[13] Autissier V; Zarza PM; Petrou A; Henderson RA; Harrington RW; Clegg WC, Proton Transfer to Nickel-Thiolate Complexes. 2. Rate-Limiting Intramolecular Proton Transfer in the Reactions of [Ni(SC6H4R-4)(PhP{CH2CH2PPh2}2)]+ (R = NO2, Cl, H, Me, or MeO). Inorg. Chem. 2004, 43 (10), 3106–3115. 15132616 10.1021/ic0303237

[14] Alwaaly A; Henderson RA, Sterics level the rates of proton transfer to [Ni(XPh){PhP(CH2CH2PPh2)2}]+ (X = O, S or Se). Chem. Commun. 2014, 50 (68), 9669–9671. 10.1039/c4cc04197f 25014682

[15] Szilagyi RK; Bryngelson PA; Maroney MJ; Hedman B; Hodgson KO; Solomon EI, S K-Edge X-ray Absorption Spectroscopic Investigation of the Ni-Containing Superoxide Dismutase Active Site: New Structural Insight into the Mechanism. J. Am. Chem. Soc. 2004, 126 (10), 3018–3019. 15012109 10.1021/ja039106v

[16] Ogata H; Nishikawa K; Lubitz W, Hydrogens detected by subatomic resolution protein crystallography in a [NiFe] hydrogenase. Nature 2015, Ahead of Print. 10.1038/nature14110 25624102

[17] Shearer J, Insight into the structure and mechanism of nickel-containing superoxide dismutase derived from peptide-based mimics. Acc. Chem. Res. 2014, 47 (8), 2332–2341. 24825124 10.1021/ar500060s

[18] Shearer J; Peck KL; Schmitt JC; Neupane KP, Cysteinate protonation and water hydrogen bonding at the active-site of a nickel superoxide dismutase metallopeptide-based mimic: Implications for the mechanism of superoxide reduction. J. Am. Chem. Soc. 2014, 136 (45), 16009–16022. 25322331 10.1021/ja5079514

[19] Shearer J; Schmitt JC; Clewett HS, Adiabaticity of the Proton-Coupled Electron-Transfer Step in the Reduction of Superoxide Effected by Nickel-Containing Superoxide Dismutase Metallopeptide-Based Mimics. J. Phys. Chem. B 2015, 119 (17), 5453–5461. 25850940 10.1021/acs.jpcb.5b02640

[20] Pelmenschikov V; Siegbahn PEM, Nickel Superoxide Dismutase Reaction Mechanism Studied by Hybrid Density Functional Methods. J. Am. Chem. Soc. 2006, 128 (23), 7466–7475. 16756300 10.1021/ja053665f

[21] Kraemer T; Kampa M; Lubitz W; van Gastel M; Neese F, Theoretical Spectroscopy of the NiII Intermediate States in the Catalytic Cycle and the Activation of [NiFe] Hydrogenases. ChemBioChem 2013, 14 (14), 1898–1905. 23703916 10.1002/cbic.201300104

[22] Shearer J, Use of a Metallopeptide-Based Mimic Provides Evidence for a Proton-Coupled Electron-Transfer Mechanism for Superoxide Reduction By Nickel-Containing Superoxide Dismutase. Angew. Chem., Int. Ed. 2013, 52 (9), 2569–2572. 10.1002/anie.201209746 PMC3752415 23341243

[23] Barondeau DP; Kassmann CJ; Bruns CK; Tainer JA; Getzoff ED, Nickel Superoxide Dismutase Structure and Mechanism. Biochemistry 2004, 43 (25), 8038–8047. 15209499 10.1021/bi0496081

[24] Wuerges J; Lee J-W; Yim Y-I; Yim H-S; Kang S-O; Carugo KD, Crystal structure of nickel-containing superoxide dismutase reveals another type of active site. Proc. Natl. Acad. Sci. U. S. A. 2004, 101 (23), 8569–8574. 15173586 10.1073/pnas.0308514101 PMC423235

[25] Ryan KC; Guce AI; Johnson OE; Brunold TC; Cabelli DE; Garman SC; Maroney MJ, Nickel Superoxide Dismutase: Structural and Functional Roles of His1 and Its H-Bonding Network. Biochemistry 2015, 54 (4), 1016–1027. 25580509 10.1021/bi501258u PMC4405897

[26] Shearer J; Long LM, A Nickel Superoxide Dismutase Maquette That Reproduces the Spectroscopic and Functional Properties of the Metalloenzyme. Inorg. Chem. 2006, 45 (6), 2358–2360. 16529443 10.1021/ic0514344

[27] Neupane KP; Shearer J, The Influence of Amine/Amide versus Bisamide Coordination in Nickel Superoxide Dismutase. Inorg. Chem. 2006, 45 (26), 10552–10566. 17173410 10.1021/ic061156o

[28] Neupane KP; Gearty K; Francis A; Shearer J, Probing Variable Axial Ligation in Nickel Superoxide Dismutase Utilizing Metallopeptide-Based Models: Insight into the Superoxide Disproportionation Mechanism. J. Am. Chem. Soc. 2007, 129 (47), 14605–14618. 17985883 10.1021/ja0731625

[29] Shearer J; Neupane KP; Callan PE, Metallopeptide Based Mimics with Substituted Histidines Approximate a Key Hydrogen Bonding Network in the Metalloenzyme Nickel Superoxide Dismutase. Inorg. Chem. 2009, 48 (22), 10560–10571. 19894770 10.1021/ic9010407 PMC2778858

[30] Tietze D; Breitzke H; Imhof D; Koeth E; Weston J; Buntkowsky G, New insight into the mode of action of nickel superoxide dismutase by investigating metallopeptide substrate models. Chem. - Eur. J. 2009, 15 (2), 517–523. 19016282 10.1002/chem.200800870

[31] Tietze D; Voigt S; Mollenhauer D; Tischler M; Imhof D; Gutmann T; Gonzalez L; Ohlenschlaeger O; Breitzke H; Goerlach M; Buntkowsky G, Revealing the Position of the Substrate in Nickel Superoxide Dismutase: A Model Study. Angew. Chem., Int. Ed. 2011, 50 (13), 2946–2950, S2946/1-S2946/22. 10.1002/anie.201005027 21404375

[32] Tietze D; Sartorius J; Koley Seth B; Herr K; Heimer P; Imhof D; Mollenhauer D; Buntkowsky G, New insights into the mechanism of nickel superoxide degradation from studies of model peptides. Sci. Rep. 2017, 7 (1), 1–15. 29222438 10.1038/s41598-017-17446-3 PMC5722923

[33] Tietze D; Koley Seth B; Brauser M; Tietze AA; Buntkowsky G, NiII Complex Formation and Protonation States at the Active Site of a Nickel Superoxide Dismutase-Derived Metallopeptide: Implications for the Mechanism of Superoxide Degradation. Chem. - Eur. J. 2018, 24 (59), 15879–15888. 30055023 10.1002/chem.201803042

[34] Shearer J; Callan PE; Amie J, Use of Metallopeptide Based Mimics Demonstrates That the Metalloprotein Nitrile Hydratase Requires Two Oxidized Cysteinates for Catalytic Activity. Inorg. Chem. 2010, 49 (19), 9064–9077. 20831172 10.1021/ic101765h PMC3570060

[35] Dutta A; Flores M; Roy S; Schmitt JC; Hamilton GA; Hartnett HE; Shearer JM; Jones AK, Sequential oxidations of thiolates and the cobalt metallocenter in a synthetic metallopeptide: Implications for the biosynthesis of nitrile hydratase. Inorg. Chem. 2013, 52 (9), 5236–5245. 23587023 10.1021/ic400171z PMC4046696

[36] Shearer J, unpublished results.

[37] Thompson PE; Keah HH; Gomme PT; Stanton PG; Hearn MT, Synthesis of peptide amides using Fmoc-based solid-phase procedures on 4-methylbenzhydrylamine resins. Int J Pept Protein Res 1995, 46 (2), 174–80. 8567172 10.1111/j.1399-3011.1995.tb01333.x

[38] Ellman GL, A colorimetric method for determining low concentrations of mercaptans. Arch Biochem Biophys 1958, 74, 443–450. 13534673 10.1016/0003-9861(58)90014-6

[39] Colpas GJ; Maroney MJ; Bagyinka C; Kumar M; Willis WS; Suib SL; Mascharak PK; Baidya N, X-ray spectroscopic studies of nickel complexes, with application to the structure of nickel sites in hydrogenases. Inorg. Chem. 1991, 30 (5), 920–8.

[40] Denny JA; Darensbourg MY, Metallodithiolates as Ligands in Coordination, Bioinorganic, and Organometallic Chemistry. Chem. Rev. 2015, 115, 5248–5273. 25948147 10.1021/cr500659u

[41] Jenkins RM; Singleton ML; Leamer LA; Reibenspies JH; Darensbourg MY, Orientation and Stereodynamic Paths of Planar Monodentate Ligands in Square Planar Nickel N2S Complexes. Inorg. Chem. 2010, 49, 5503–5514. 20507173 10.1021/ic1002012 PMC2902169

[42] Neese F, Prediction of molecular properties and molecular spectroscopy with density functional theory: From fundamental theory to exchange-coupling. Coord. Chem. Rev. 2009, 253 (5+6), 526–563.

[43] Kirchner B; Wennmohs F; Ye S; Neese F, Theoretical bioinorganic chemistry: the electronic structure makes a difference. Curr. Opin. Chem. Biol. 2007, 11 (2), 134–141. 17349817 10.1016/j.cbpa.2007.02.026

[44] Jenkins RM; Singleton ML; Almaraz E; Relbenspies JH; Darensbourg MY, Imidazole-Containing (N3S)-Ni-II Complexes Relating to Nickel Containing Biomolecules. Inorg. Chem. 2009, 48, 7280–7293. 19572492 10.1021/ic900778k PMC2908898

[45] Grapperhaus CA; Darensbourg MY, Oxygen Capture by Sulfur in Nickel Thiolates. Acc. Chem. Res. 1998, 31 (8), 451–459.

[46] Green KN; Brothers SM; Jenkins RM; Carson CE; Grapperhaus CA; Darensbourg MY, An Experimental and Computational Study of Sulfur-Modified Nucleophilicity in a Dianionic NiN2S2 Complex. Inorg. Chem. 2007, 46 (18), 7536–7544. 17685511 10.1021/ic700878y

[47] Shearer J; Dehestani A; Abanda F, Probing Variable Amine/Amide Ligation in NiIIN2S2 Complexes Using Sulfur K-Edge and Nickel L-Edge X-ray Absorption Spectroscopies: Implications for the Active Site of Nickel Superoxide Dismutase. Inorg. Chem. 2008, 47 (7), 2649–2660. 18330983 10.1021/ic7019878

[48] Mullins CS; Grapperhaus CA; Kozlowski PM, Density functional theory investigations of NiN2S2 reactivity as a function of nitrogen donor type and N-H···S hydrogen bonding inspired by nickel-containing superoxide dismutase. J. Biol. Inorg. Chem. 2006, 11 (5), 617–625. 16724228 10.1007/s00775-006-0109-6

[49] Mullins CS; Grapperhaus CA; Frye BC; Wood LH; Hay AJ; Buchanan RM; Mashuta MS, Synthesis and Sulfur Oxygenation of a (N3S)Ni Complex Related to Nickel-Containing Superoxide Dismutase. Inorg. Chem. 2009, 48 (21), 9974–9976. 19795870 10.1021/ic901246w

[50] Herdt DR; Grapperhaus CA, Kinetic study of nickel-thiolate oxygenation by hydrogen peroxide. Implications for nickel-containing superoxide dismutase. Dalton Trans. 2012, 41 (2), 364–366. 22105369 10.1039/c1dt11300c

[51] Martinage O; Clainche LLC,B; Dugave C, Synthesis and biological evaluation of a new triazole-oxotechnetium complex. Org. Biomol. Chem. 2012, 10, 6484–6490. 22752052 10.1039/c2ob25774b

[52] Neese F, The ORCA program system. Wiley Interdiscip. Rev.: Comput. Mol. Sci. 2012, 2 (1), 73–78.

[53] Weigend FA, Reinhart, Balanced basis sets of split valence, triple zeta valance and quadruple zeta valence quality for H to Rn: Design and assessment of accuracy. Phys. Chem. Chem. Phys. 2005, 7, 3297–3305. 16240044 10.1039/b508541a

[54] Grimme SE, Stephan; Goerigk, Lars, Effect of the damping function in dispersion corrected density functional theory. J. Comput. Chem. 2011, 32, 1456–1465. 21370243 10.1002/jcc.21759

[55] Grimme SA, Jens; Ehrlich, Stephan; Krieg, Helge, A consistent and accurate ab initio parameterization of density functional dispersion correction (DFT-D) for the 94 elements H-Pu. J. Chem. Phys. 2010, 132, 154104. 10.1063/1.3382344 20423165

[56] Hellweg AH,C; Hofener S; Klopper W, Optimized accurate auxiliary basis sets for RI-MP2 and RI-CC2 calculations for the atoms Rb to Rn. Theor. Chem. Acc. 2007, 117, 587.

[57] Weigend F, Accurate Coulomb-fitting basis sets for H to Rn. Chem. Phys. Phys. Chem. 2006, 8, 1057–1065. 10.1039/b515623h 16633586

[58] Pettersen EFG,TD; Huang CC; Couch GS; Greenblatt DM; Meng EC; Ferrin TE, UCSF Chimera--a visualization system for exploratory research and analysis. J. Comput. Chem. 2004, 25, 1605–1612. 15264254 10.1002/jcc.20084

