# Supplementary material for: pH Dependent Reversible Formation of a Binuclear Ni2 Metal-Center Within a Peptide Scaffold
Source: Inorganics (Basel). Author manuscript; Available in PMC 2023 Dec 1. (PMC10691859; doi:10.3390/inorganics7070090)
Supplement: SI [file NIHMS1055816-supplement-SI.docx]

**Supporting Information for:**

**Multiple Cysteinate Protonation At the Metal-Site of a Ni^II^NS_3_ Metallopeptide – Implications For Cysteinate-Ligated Biological Nickel Centers**

Jennifer C. Schmitt^‡^, Brenna C. Keegan^‡^, Tyler Detomasi, and Jason Shearer*

*Department of Chemistry, University of Nevada, Reno, Reno, NV 89557, USA*

**Contains:**

1) Experimental Procedures

**Experimental**

**General Considerations.** All manipulations were performed under an N_2_/H_2_ (97:3) atmosphere in a COY anaerobic chamber. Fmoc/O*^t^*Bu protected amino acids and resins were obtained from Advanced Chemtech (Louisville, KY). All other reagents obtained from commercial suppliers were of the highest purity available, and used as received. Analytical and semi-preparative reverse-phase HPLC were performed using Waters X-Bridge C-18 analytical (4.6 × 150 mm; 5 μm) and semi-preparative (30 × 150 mm; 5 μm) columns on a Waters Deltaprep 600 equipped with a photodiode array detector (detection wavelength set to 254 nm). Mass specrtrometry was performed on either a Bruker Microflex MALDI-TOF mass spectrometer or a Waters Micromass 20 ESI-MS operating in positive ion mode. NMR spectra were obtained on a 400 MHz Varian VNMRS NMR spectrometer. All chemical shifts (*δ*) are referenced to the residual solvent peak.

**Preparation of S-triphenylmethyl-thioacetic acid.** S-triphenylmethyl-mercatoacetic acid (T^A^-trityl) was prepared by a modification of the procedure on Martinage et al (Org. Biomol. Chem. 2012, 10, 6484). Briefly, 1.5 g (5.77 mmol) of triphenylmethanol was added to a 25 mL of a TFA solution of 2-mercapto-acetic acid (400 μL, 5.77 mmol) and stirred for 5 h under argon at room temperature. The TFA was removed under vacuum, and the resulting orange solid was washed three times with toluene followed by three times with hexanes resulting in a white solid, which was analytically pure (1.46 g, 81% yield). ^1^H NMR (CDCl_3_, 400 MHz): *δ* 7.30 (m, 15H), 3.04 (s, 2H).

**Preparation of {Ni(m1S_3_)}.** The peptide m1S_3_ (T^a^CDLP-CGVYD-PA) was prepared on an AAPTec Focus XC-2RV peptide synthesizer using HBTU/HOBt coupling strategies on alanine loaded Wang resin (0.1 mmole scale) using a five-fold excess of activated protected amino acid. Following the coupling trityl protected Cys(2) and removal of the Fmoc group, T^A^-trityl was coupled to the *N*-terminus using standard HBTU/HOBt coupling strategies. Global peptide deprotection and peptide cleavage from the resin was performed under N_2_ using a cleavage cocktail comprised of TFA/EDT/thioanisol/phenol/water (82.5:2.5:5:5:5) over the course of 14 hours. Following removal of the cleavage solution by vacuum on a Schlenk line, the resulting glassy product was washed four times with cold freshly distilled diethyl ether. The resulting crude peptide was subsequently purified by preparative HPLC (10 – 29% MeCN (0.1% TFA) in water (0.1% TFA); rt = 12.10 min) resulting in 15 mg of the pure peptide m1S_3_ (12% yield). Analytical HPLC: 10 – 29% MeCN (0.1% TFA) in water (0.1% TFA) (rt = 21.05 min). MALDI-TOF MS: m/z calcd. For (m1S_3_+Na)^+^ 1249 Da, found 1249 Da.

Solutions of m1S_3_ in 50 mM NEM buffer (pH 7.4 or 9.5) were prepared and 1.0 equiv of NiCl_2_ (added from a pH 7.0 50 mM stock solution) were then added to solutions of the m1S_3_. The number of free thiol groups per peptide was verified using an Ellman’s assay compared to the peptide concentration as determined by the absorbance of the Y residue (λ_max_ = 278 nm; ε = 1,490 M^–1^ cm^–1^). ESI-MS data were obtained by injecting an air-free solutions of the metallopeptide into the mass spectrometer using an air-tight syringe (ESI-MS: calcd.{Ni^II^(m1S_3_)}+Na)^+^ 1307 Da, found 1307 Da.). Air oxidation of {Ni^II^(m1S_3_)} was accomplished by exposing solutions of the metallopeptide at pH 7.4 or 9.5 to air for 12 hours. CD spectroscopy (ε (cm^–1^) (Δε M^–1^ cm^–1^)): MALDI-MS: m/z calcd. ({Ni^II^(m1S_3_)}+Na)^+^

**Determination of p*K_a_* and *K_d_* values.** Solutions of {Ni^II^(m1S_3_)} were dissolved in 50 mM NEM buffer at a pH of 6.5. Small aliquots of NaOH were then added to the solution. Solution pH was monitored using a pH microelectrode and the electronic absorption measured following each addition. The resulting pH titration curve was constructed by monitoring the change in absorbance at λ = 400 nm. The pH values were then obtained by determining the inflection points of the pH titration curve. Dissociation constants at pH 7.4, 8.0 and 9.6 were obtained by competitive glycine (pH 7.0, 7.4, and 8.0) or lysine (pH 9.6) binding assays as previously described.

**Kinetics of the Air Oxidation of {Ni^II^(m1S_3_)}.** The CD spectrum of anaerobically prepared solutions of {Ni^II^(m1S_3_)} at pH 7.4 or 9.6 (50 mM NEM buffer) were recorded and then air was bubbled through the metallopeptide solutions for five min. A CD spectrum of these solutions was subsequently taken every 10 min for 12 hours with the sample continuously exposed to air. The oxidation kinetics was modeled using pseudo first reaction kinetics using *KinTek Explorer v 5.2*.

**Electronic Absorption and CD Spectroscopy.** Electronic absorption and CD spectra were obtained at a pH of 7.4 or 9.6 (50 mM NEM buffer) in 1 cm quartz cuvets at 20 °C. Electronic absorption spectra were obtained on a Perkin Elmer Lambda 700 spectrometer while CD spectra were obtained on a JACSO 1500 spectropolarimeter. The electronic absorption and CD spectra were simultaneously deconvolved into the minimum number of Gaussian line shapes using the program *Igor Pro* v. 6.37.

**Nickel K-edge X-ray Absorption Spectroscopy.** Nickel K-edge X-ray absorption spectroscopic data were collected on beamline X-3b at the National Synchrotron Light Source (Upton, NY, USA) and the HXMA beamline at the Canadian Light Source (Saskatoon, SA, Canada). Solutions of {Ni^II^(m1S_3_)} (1.0 mM in 1:1 50 mM NEM buffer:glycerol at a pH of 7.4 or 9.5) were injected between Kapton tape windows in aluminum sample holders and quickly frozen in liquid nitrogen. Data were collected at 20 K with sample temperatures maintained using either a He displex cryostat (X-3b) or an Oxford liquid He flow cryostat (HXMA). On X-3b light was monochromatized using a Si(111) double crystal monochromator and focused using a low angle nickel mirror, which was also employed for harmonic rejection. On the HXMA line light was monochromatized using a Si(220) double crystal monochromator, which was detuned 50% for harmonic rejection, and focused using a Rh mirror. Despite the differences in beamline optics and insertion devices (bending magnet on X-3b vs wiggler operating at 1.5 T on HXMA), the data quality obtained on the two lines were comparable. Spectra were obtained in fluorescence mode using a 32 element solid-state Ge detector on both lines with a 3 micron cobalt filter placed between the sample and detector, and spectra were calibrated against the first inflection point of Ni-foil, which was simultaneously recorded with the metallopeptide data. Data were obtained in 10 eV steps in the pre-edge region (8130 – 8300 eV, 1 s integration time), 0.3 eV steps in the pre-edge region (8300 – 8350 eV, 2 s integration time), 1.0 eV steps in the edge region (8350 – 8400 eV, 2 s integration time), 2.0 eV steps in the near edge region (8400 – 8640 eV, 3 s integration time), and 0.05 k steps in the far edge region (8640 eV – 16.0 Å^–1^, 3 s integration time). Total fluorescence counts were maintained under 30 kHz, and a deadtime correction yielded no appreciable change to the data. The reported spectra represent the averaged spectra from 5 individual data sets. Prior to data averaging each spectrum and detector channel was individually inspected for data quality. Although data were recorded to 16 Å^–1^, the data were analyzed only to 15.0 Å^–1^ owing to noise at high *k*. Data were subsequently processed and analyzed as previously reported using *EXAFS123* and FEFF 9.4. Errors to the models are reported as ε^2^ values (REF).

**Sulfur K-edge X-ray Absorption Spectroscopy.** Solutions of {Ni(m1S_3_)} were prepared at a pH of either 7.4 or 9.5 (~1 mM in 50 mM NEM buffer) and injected into lucite sample holders with polypropylene windows. Data were obtained at room temperature (~20 °C) on beamline X-19a at the NSLS (Upton, NY) in a He purged sample chamber using a passivated implanted planar silicon (PIPS) detector. The photon energies were calibrated against the first inflection point of S_8_ recorded before and after each sample; it was found that there was no detectable monochromator drift throughout the data collection. Data were obtained in 5 eV steps in the pre-edge region (2250 – 2460 eV), 0.1 eV steps in the edge region (2460 – 2482 eV) and 2 eV steps in the post edge region (2482 – 2800 eV). The reported data represents the average of five individual scans. Following data averaging and a baseline was applied to each spectrum by fitting the pre-edge region to a polynomial function. This baseline was then subtracted from the whole spectrum. The region above the edge jump was then fit to a two-knot cubic spline, and the data normalized to the edge height. Pre-edge and the rising-edge features were modeled as pseudo-Voigt line shapes (a 1:1 sum of Gaussian and Lorentzian line shapes). Each spectrum required the use of only one peak per feature, and valid fits to the data were judged by matches to the second derivatives of the spectra. The intensity values of the pre-edge features, which represent the average of four different fits to the data (differing by less than 2%), are the products of the peak widths at half- height and intensities of the pseudo-Voigt line shapes. All pre-edge intensities were then converted into %S3p character using the pre-edge feature of Ni(DACO) as a standard (46(2)%), which by our fitting procedure corresponds to an intensity of 1.22(1) units.

**Electronic Structure Calculation.** Electronic structure calculations were performed using *ORCA* v 3.03. Unless otherwise stated, all calculations employed Ahlrichs’ def2-tzvp basis set on all atoms and the atom pairwise dispersion correction with Becke-Johnson damping to account for dispersive interactions. ORCA VeryTightSCF convergence criteria were used for the SCF cycles, with program defaults used for all other convergence criteria and settings. Geometry optimizations were performed at the BP86 level, and used the RI approximation and def2-tzvp/c auxiliary basis set. Single point calculations were performed at the PBE0 level and used the RIJCOSX approximation and def2-tzvp/j auxiliary basis set. Truncated models of {Ni^II^(m1S_3_)} were constructed by bonding Ni(II) to deprotonated 2-mercapto-*N*-(2-mercaptoethyl)acetamide and an ethane thiolate (Chart X). The models contained either no deprotonated sulfur atoms (NiS_3_), one protonated sulfur atom (NiS_2_(SH^+^)), or two protonated sulfur atoms (NiS(SH^+^)_2_, Figure X). Electronic absorption spectra were simulated by calculating the transition energies and oscillator strengths of the first 50 transitions using standard TD-DFT methods (PBE0/def2-tzvp). In addition, the first five low energy transitions were calculated using the spectroscopically oriented CI (SORCI) method. These calculations utilized a CAS-SCF(8,8) active space…. A XXX cm^–1^ red shift was applied to each transition. A Gaussian function to each transition (FWHM = 1200 cm^–1^), and summing the individual transitions. Nickel and sulfur K-edge X-ray absorption spectra were simulated using DFT/ROCIS calculations (B3LYP/def2-tzvp(-f) and the ZORA relativistic approximation) with appropriate scaling of the coulomb (c_1_ = 0.21) and exchange (c_2_ = 0.49) parts of the CI matrix and a reduction in the off-diagonal elements (c_3_ = 0.29) of the CI matrix. The first 25 root originating from sulfur or the nickel 1s orbital were calculated. A Gaussian function to each transition (FWHM = 0.75 eV for S K-edge calculations and 1.2 eV for Ni K-edge calculations) followed by summing the individual transitions. A +41.1 eV (sulfur K-edge) or +9.0 eV (nickel K-edge) energy correction was applied to each transition to best match the experimental S or Ni K-edge data. Atomic orbital population analyses were performed using a Löwdin population analysis. Isosurface plots were generated using Chimera v 1.1.
