# Supplementary material for: pH Dependent Reversible Formation of a Binuclear Ni2 Metal-Center Within a Peptide Scaffold
Source: Inorganics (Basel). Author manuscript; Available in PMC 2023 Dec 1. (PMC10691859; doi:10.3390/inorganics7070090)
Supplement: Table S2 [file NIHMS1055816-supplement-Table_S2.pdf]

Table S2. Cartesian coordinates for monoprotonated mononuclear computational model 1

Ni	-2.762083	0.095212	-0.091059
S	-1.502386	-1.652619	-0.326083
N	-3.895584	1.629494	0.089158
S	-4.457719	-1.187008	0.183132
C	-5.210483	1.359322	0.693197
C	-5.769217	0.086897	0.090943
H	-6.059257	0.248483	-0.957489
H	-6.645049	-0.271147	0.650939
C	-1.469965	-1.879362	-2.154619
H	-2.514057	-1.870058	-2.490878
C	-0.721738	-3.137750	-2.570713
H	-0.982321	-0.963241	-2.512750
H	-5.083619	1.219427	1.780602
H	-5.905409	2.197520	0.535493
H	-0.685637	-3.202184	-3.668358
H	-1.221517	-4.043316	-2.198689
H	0.309745	-3.135021	-2.194201
C	-3.559665	2.866016	-0.250412
C	-4.515401	4.033835	-0.098868
O	-2.401791	3.163920	-0.730727
H	-5.425780	3.893057	-0.697942
H	-4.001024	4.936894	-0.442169
H	-4.820790	4.171303	0.947949
O	-1.137771	1.121474	-0.506115
H	-0.596651	1.185982	0.301023
H	-1.664423	2.150421	-0.656203
H	-2.303650	-2.722333	-0.066321
