# Supplementary material for: pH Dependent Reversible Formation of a Binuclear Ni2 Metal-Center Within a Peptide Scaffold
Source: Inorganics (Basel). Author manuscript; Available in PMC 2023 Dec 1. (PMC10691859; doi:10.3390/inorganics7070090)
Supplement: Table S3 [file NIHMS1055816-supplement-Table_S3.pdf]

Table S3. Cartesian coordinates for monoprotonated mononuclear computational model 2

Ni	-2.576261	0.109883	-0.264504
S	-1.110795	-1.535403	-0.185145
N	-3.916314	1.513468	-0.082218
S	-3.932188	-1.298937	0.509338
C	-5.160267	1.103673	0.579411
C	-5.488633	-0.333434	0.209166
H	-5.735932	-0.416555	-0.855620
H	-6.302288	-0.746429	0.819746
C	-0.814051	-1.850625	-1.969926
H	-0.484089	-0.913377	-2.438164
C	-2.020430	-2.424347	-2.706980
H	0.030491	-2.556949	-1.997577
H	-5.060743	1.182995	1.678959
H	-6.011795	1.738523	0.287583
H	-1.767970	-2.660228	-3.752678
H	-2.844952	-1.695645	-2.715294
H	-2.374445	-3.346189	-2.223028
C	-3.694029	2.814376	-0.284766
C	-4.741067	3.854570	0.070420
O	-2.605694	3.257002	-0.784434
H	-5.653700	3.723457	-0.529274
H	-4.323411	4.844603	-0.136951
H	-5.028818	3.797162	1.130033
O	-1.295344	1.238859	-1.132775
H	-0.458454	1.136707	-0.644219
H	-1.762071	2.228692	-0.983709
H	-4.073319	-2.178250	-0.516574
