# Supplementary material for: pH Dependent Reversible Formation of a Binuclear Ni2 Metal-Center Within a Peptide Scaffold
Source: Inorganics (Basel). Author manuscript; Available in PMC 2023 Dec 1. (PMC10691859; doi:10.3390/inorganics7070090)
Supplement: Table S1 [file NIHMS1055816-supplement-Table_S1.pdf]

Table S1. Cartesian coordinates for diprotonated mononuclear computational model

Ni	-2.985135	-0.031619	-0.178560
S	-1.743194	-1.825451	-0.429911
N	-3.964080	1.600612	0.102848
S	-4.817341	-1.129247	0.170119
C	-5.190115	1.433327	0.893085
C	-5.999930	0.285683	0.314604
H	-6.374382	0.518530	-0.687922
H	-6.824953	-0.027229	0.965429
C	-1.320174	-1.887424	-2.226242
H	-2.255814	-1.925073	-2.796327
C	-0.394208	-3.055879	-2.530821
H	-0.845038	-0.912057	-2.390968
H	-4.922151	1.218433	1.940665
H	-5.804520	2.343287	0.898201
H	-0.119310	-3.020468	-3.594487
H	-0.876729	-4.024608	-2.342371
H	0.528279	-3.003284	-1.939663
C	-3.607632	2.797631	-0.337038
C	-4.452905	4.031275	-0.176841
O	-2.478352	2.982798	-0.946625
H	-5.476535	3.882721	-0.545724
H	-3.983760	4.842709	-0.741389
H	-4.509892	4.328984	0.880340
O	-1.360277	0.838382	-0.591012
H	-0.801696	0.887859	0.207924
H	-1.880857	2.073105	-0.856301
H	-5.145513	-1.523389	-1.090133
H	-2.542567	-2.927757	-0.402168
