# Supplementary material for: pH Dependent Reversible Formation of a Binuclear Ni2 Metal-Center Within a Peptide Scaffold
Source: Inorganics (Basel). Author manuscript; Available in PMC 2023 Dec 1. (PMC10691859; doi:10.3390/inorganics7070090)
Supplement: Table S4 [file NIHMS1055816-supplement-Table_S4.pdf]

Table S4. Cartesian coordinates for unprotonated mononuclear computational model

Ni	-2.621850	0.211296	-0.439613
S	-0.974827	-1.254743	-0.319281
N	-3.945452	1.618635	-0.175455
S	-3.996350	-1.264124	0.281620
C	-5.225326	1.177543	0.400506
C	-5.501469	-0.253219	-0.015595
H	-5.768533	-0.287538	-1.084201
H	-6.342056	-0.670427	0.563637
C	-0.808021	-1.808964	-2.066248
H	-0.803823	-0.920209	-2.714797
C	-1.904974	-2.781142	-2.492036
H	0.185431	-2.283174	-2.147620
H	-5.176040	1.218476	1.506801
H	-6.056649	1.833396	0.078260
H	-1.790985	-3.077404	-3.550788
H	-2.892244	-2.318064	-2.351152
H	-1.884328	-3.686248	-1.868162
C	-3.671345	2.919999	-0.163144
C	-4.692178	3.919297	0.377652
O	-2.569673	3.427953	-0.576873
H	-5.597537	3.948790	-0.247932
H	-4.228945	4.912310	0.380854
H	-5.013313	3.661810	1.398436
O	-1.353100	1.421163	-1.376691
H	-0.547181	1.127692	-0.901680
H	-1.720093	2.348774	-1.020410
