# Supplementary material for: pH Dependent Reversible Formation of a Binuclear Ni2 Metal-Center Within a Peptide Scaffold
Source: Inorganics (Basel). Author manuscript; Available in PMC 2023 Dec 1. (PMC10691859; doi:10.3390/inorganics7070090)
Supplement: Table S5 [file NIHMS1055816-supplement-Table_S5.pdf]

Table S5. Cartesian coordinates for dinuclear computational model with disulfide bridge

Ni	-2.51490301364867	0.64197685369813	-0.17096724008139
Ni	0.74014313849295	0.98469720182281	-0.25947574875621
S	-0.98015818158893	2.30225752472681	-0.39900052038534
S	-0.78388734027928	-0.71981870935281	-0.43109972474445
N	-3.89820352011724	1.81949405065690	0.41418993900667
N	2.20558142116705	0.02681583049738	0.63557993109239
S	-4.14330122753410	-0.71650533789512	-0.83818908936127
S	2.22692530428104	2.45459092517140	-0.97327623272906
C	-5.20830375941819	1.75411807337605	-0.23218757691469
C	-5.16182489046856	0.72439281506730	-1.35627006098373
H	-4.71676265524132	1.16956371690929	-2.26109203348955
H	-6.18064388107189	0.38579521647507	-1.61388440628336
C	3.57563816503567	0.39323461203726	0.24272283541414
C	3.55971134525440	1.18715460743187	-1.05291953587303
H	3.37704205570846	0.52244784789456	-1.91360307958985
H	4.53219291353725	1.68633726119669	-1.20978088316548
C	-3.86325515178102	2.42286682406089	1.61280192114590
C	-2.55952941536209	2.24643745040331	2.41368562965242
C	2.13961128984192	-0.49545890532285	1.87685180144483
C	0.75757611141730	-0.77534205762877	2.47831166955725
C	-1.32022940919935	3.05796849883591	-2.03679315879005
H	-2.26249620258012	3.61290991468094	-1.90194946149018
C	-1.42712281732474	2.06745365940994	-3.18537724159220
H	-0.50731691451799	3.77831849090676	-2.21755363062377
C	-0.46022279602306	-1.25286405245008	-2.16149019636058
H	-0.58365892875014	-0.39054162018782	-2.82649366532772
C	-1.37807030281665	-2.40020494908069	-2.57340484728163
H	0.60106597281388	-1.54580475367127	-2.18552178496583
S	-2.03352780733485	0.49348804505957	2.35590539739753
H	-2.75742111462946	2.55237207998390	3.44716633511636
H	-1.74954960155561	2.85242787886582	1.97951598401016
S	-0.06673637323812	0.70185457513264	3.22483443438733
H	0.89559694366733	-1.50936045028911	3.28200912054422
H	0.07060128961083	-1.17370924886978	1.71955016177179
H	4.03434808320471	0.99661193638393	1.04788658527556
H	4.20284865899031	-0.51523477319337	0.14372455133098
H	-5.96618360675445	1.47501859387065	0.52238244589159
H	-5.50716238839299	2.74760718053940	-0.62293716667775
O	3.14575405743770	-0.78398016527726	2.57962309280428
O	-4.80107896098473	3.08800660308412	2.11823792898870
H	-2.15798061947266	1.28110897007692	-2.93886176952113
H	-0.44989921637217	1.59322818937128	-3.35331965772429
H	-1.73876562368148	2.56844681662362	-4.11977785422421
H	-1.16616579037055	-2.71830674391743	-3.61019372403252
H	-2.43036809087872	-2.08404870954646	-2.50050082080092
H	-1.24069714907167	-3.26766176756834	-1.90981865306189
