# Supplementary material for: pH Dependent Reversible Formation of a Binuclear Ni2 Metal-Center Within a Peptide Scaffold
Source: Inorganics (Basel). Author manuscript; Available in PMC 2023 Dec 1. (PMC10691859; doi:10.3390/inorganics7070090)
Supplement: Table S6 [file NIHMS1055816-supplement-Table_S6.pdf]

Table S6. Cartesian coordinates for dinuclear computational model without disulfide bridge

Ni	-1.95724154983937	0.41587677611011	0.77096677378535
Ni	1.41117325675870	0.61228594823025	0.76961751704832
S	-0.34760385247717	1.96014566719011	0.67532962844414
S	-0.19811229781733	-0.93423195505679	0.69849162018455
C	-0.43770721873177	2.45829163066570	-1.10791119537641
C	0.39608721925621	3.70112274411391	-1.39799639431977
H	-1.50490575418095	2.61500183642790	-1.32177839084123
H	-0.09191644066321	1.60583366982723	-1.70824284674929
H	0.02713036187950	4.55997939936934	-0.81654120851186
H	0.34889251343151	3.96191045737697	-2.47087652727604
H	1.44542851787835	3.52446246432583	-1.11379427128554
C	-0.10662218825057	-1.45856081877585	-1.07711128435693
C	-0.95032323641582	-2.69730845328761	-1.35498444513928
H	0.96010109192605	-1.62857761227082	-1.28403854043988
H	-0.44032040152642	-0.61115595438840	-1.69137429611131
H	-0.59124513736368	-3.55205943739428	-0.76135812986570
H	-0.90114905619715	-2.97203416387785	-2.42423211725175
H	-1.99913559924521	-2.50892138833322	-1.07645427854543
N	-3.48190463727620	1.61117659382546	0.75523652693466
C	-3.38172652146058	3.06657772778493	0.77275371410761
H	-2.34977099536056	3.35875235154982	1.00780362439355
H	-4.06593192072265	3.50157504348641	1.52209501452548
H	-3.66686346679785	3.51202070425471	-0.20052521956206
N	2.93601823253994	-0.58256474493317	0.77473274139279
C	2.83792395691288	-2.03735209797209	0.82458929598586
H	1.80375483246188	-2.32578228156498	1.05468869191357
H	3.51377947925314	-2.45400706089037	1.59194604476226
H	3.13549454556174	-2.50442454768404	-0.13463200801790
S	2.80743792594696	2.29410038403829	0.87965019241555
C	4.37657689009744	1.37832137891309	0.68503183317147
C	4.20778899567022	-0.13675894912601	0.69991772953657
H	5.07992748737457	1.64057911055507	1.49176308454332
H	4.86433003525908	1.65023999393693	-0.26563366719926
S	-3.35421548838054	-1.26279991515597	0.91321453065065
C	-4.92408896617903	-0.34989746600857	0.71122514635264
C	-4.75414258740132	1.16497326613674	0.69140122557518
H	-5.62106912595636	-0.59348184412738	1.52936015466862
H	-5.41974300503734	-0.64241389018354	-0.22911159274674
O	5.22972086857331	-0.86976839344558	0.64585953744975
O	-5.77565676350040	1.89731382635771	0.62205178575444
