# Supplementary material for: pH Dependent Reversible Formation of a Binuclear Ni2 Metal-Center Within a Peptide Scaffold
Source: Inorganics (Basel). Author manuscript; Available in PMC 2023 Dec 1. (PMC10691859; doi:10.3390/inorganics7070090)
Supplement: Table S7 [file NIHMS1055816-supplement-Table_S7.pdf]

Table S7. Cartesian coordinates for protonated dinuclear computational model with disulfide bridge

Ni	-2.45974	0.52932	-0.02581
Ni	0.74013	0.98796	-0.14261
S	-0.91908	2.41076	-0.42043
S	-0.65782	-0.72324	-0.31182
N	-3.88712	1.73842	0.42032
N	2.20225	0.05143	0.67054
S	-4.23109	-0.83340	-0.27424
S	2.16302	2.32165	-1.09860
C	-5.10591	1.72223	-0.37494
C	-5.11696	0.46299	-1.24917
H	-4.54437	0.61104	-2.17309
H	-6.13684	0.13188	-1.48971
C	3.56332	0.33659	0.18539
C	3.48430	1.03799	-1.15964
H	3.25006	0.31954	-1.96026
H	4.44065	1.52695	-1.40622
C	-3.92942	2.41520	1.58596
C	-2.68416	2.27200	2.47713
C	2.16048	-0.48813	1.90577
C	0.77649	-0.64596	2.57243
C	-1.27080	3.19162	-2.04330
H	-2.26839	3.64308	-1.93562
C	-1.19308	2.26958	-3.24653
H	-0.53182	4.00324	-2.12567
C	-0.22860	-1.00856	-2.07414
H	0.13139	-0.06300	-2.49662
C	-1.37677	-1.58614	-2.89166
H	0.62732	-1.69986	-2.04402
S	-2.06090	0.54893	2.50774
H	-2.94035	2.60561	3.48791
H	-1.87567	2.89520	2.06300
S	-0.01424	0.97270	2.93543
H	0.91847	-1.19124	3.51154
H	0.10004	-1.19817	1.90651
H	4.08228	0.97844	0.92001
H	4.14929	-0.59774	0.11624
H	-5.97963	1.75011	0.29807
H	-5.18651	2.60925	-1.03083
O	3.16083	-0.88096	2.54667
O	-4.87339	3.13382	1.96701
H	-1.86395	1.40932	-3.10968
H	-0.16629	1.89954	-3.36550
H	-1.48238	2.80010	-4.16919
H	-1.05734	-1.80637	-3.92298
H	-2.20821	-0.86686	-2.94060
H	-1.75405	-2.51689	-2.44206
H	-3.97670	-1.66685	-1.31603
