# Supplementary material for: pH Dependent Reversible Formation of a Binuclear Ni2 Metal-Center Within a Peptide Scaffold
Source: Inorganics (Basel). Author manuscript; Available in PMC 2023 Dec 1. (PMC10691859; doi:10.3390/inorganics7070090)
Supplement: Table S8 [file NIHMS1055816-supplement-Table_S8.pdf]

Table S8. Alternative EXAFS models for {Ni(SOD^{mds})} at pH 7.4.[a]

	Reported	Best Fit[b]	S_3N	S_2NO	S_2N_2Ni
**Shell #1 Ni-S**					
N	2	2.36(13)	3	2	2
R (Å)	2.1804(14)	2.1737(15)	2.180(1)	2.1822(14)	2.174(2)
σ^2 (Å^2)	0.0026(2)	0.0038(3)	0.0050(2)	0.00026(20)	0.0032(14)
**Shell #2 Ni-N**					
N	2	1.6(7)	1	1	2
R (Å)	1.907(16)	1.893(18)	1.823(11)	1.802(6)	1.912(11)
σ^2 (Å^2)	0.0013(6)	0.0063(7)	0.0082(15)	0.0053(6)	0.006(2)
**Shell #3 Ni-O**					
N				1	
R (Å)	N/A	N/A	N/A	1.935(6)	N/A
σ^2 (Å^2)				0.0006(6)	
**Shell #4 Ni-Ni**					
N					1
R (Å)	N/A	N/A	N/A	N/A	2.62(5)
σ^2 (Å^2)					0.017(2)
**E_o (eV)**	8347.1	8447.3	8446.8	8447.5	8447.2
**ε^2**	0.69	0.61	0.80	0.67	0.65

[a] All values for the number of shells was restrained to the nearest whole number. [b] The best fit (i.e. lowest ε^2) allowed the number of scatterers in each shell to refine.
