# Supplementary material for: pH Dependent Reversible Formation of a Binuclear Ni2 Metal-Center Within a Peptide Scaffold
Source: Inorganics (Basel). Author manuscript; Available in PMC 2023 Dec 1. (PMC10691859; doi:10.3390/inorganics7070090)
Supplement: Table S9 [file NIHMS1055816-supplement-Table_S9.pdf]

Table S9. Alternative EXAFS models for {Ni(SOD^{mds})} at pH 9.6.[a]

	**Reported**	**Best Fit**[b]	**S_3N**	**S_2N_2**	**S_2N_2Ni**
**Shell #1 Ni-S**					
N	3	2.8(4)	2	2	2
R (Å)	2.229(2)	2.202(2)	2.200(2)	2.203(2)	2.204(2)
σ^2 (Å^2)	0.0044(2)	0.0019(5)	0.00012(2)	0.00014(2)	0.00012(2)
**Shell#2 Ni-S**					
N			1		
R (Å)	N/A	N/A	2.5(2)	N/A	N/A
σ^2 (Å^2)			0.06(7)		
**Shell #3 Ni-N**					
N	1	1.2(4)	1	2	2
R (Å)	1.889(9)	1.886(11)	1.926(8)	1.935(8)	1.894(8)
σ^2 (Å^2)	0.0013(8)	0.003(2)	0.0009(8)	0.0053(11)	0.0094(11)
**Shell #4 Ni-Ni**					
N	1	0.7(2)			1
R (Å)	3.25(3)	3.219(10)	N/A	N/A	3.21(3)
σ^2 (Å^2)	0.0061(15)	0.006(2)			0.0075(15)
**E_o (eV)**	8346.4	8346.2	8346.8	8346.2	8346.5
**ε^2**	1.47	1.01	1.56	1.81	1.77

[a] All values for the number of shells was restrained to the nearest whole number. [b] The best fit (i.e. lowest ε^2) allowed the number of scatterers in each shell to refine.
