# Supplementary figures and images for: pH Dependent Reversible Formation of a Binuclear Ni2 Metal-Center Within a Peptide Scaffold
Source: Inorganics (Basel). Author manuscript; Available in PMC 2023 Dec 1. (PMC10691859; doi:10.3390/inorganics7070090)

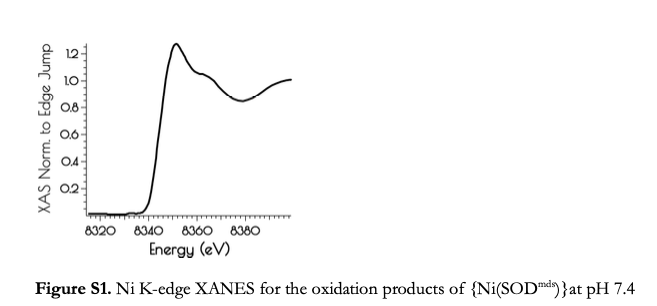

Supplement: Fig S1 [file NIHMS1055816-supplement-Fig_S1.png]

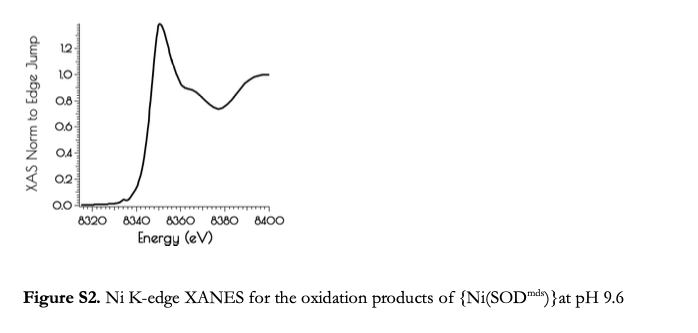

Supplement: FigS2 [file NIHMS1055816-supplement-FigS2.png]
